# Association of longitudinal platelet count trajectory with ICU mortality: A multi-cohort study

**DOI:** 10.3389/fimmu.2022.936662

**Published:** 2022-08-19

**Authors:** Jiajin Chen, Xi Gao, Sipeng Shen, Jingyuan Xu, Zhe Sun, Ruilang Lin, Zhixiang Dai, Li Su, David C. Christiani, Feng Chen, Ruyang Zhang, Yongyue Wei

**Affiliations:** ^1^ Department of Biostatistics, Center for Global Health, School of Public Health, Nanjing Medical University, Nanjing, China; ^2^ Department of Immunology, School of Clinical Medicine, Nanjing Medical University, Nanjing, China; ^3^ Department of Critical Care Medicine, Zhongda Hospital, School of Medicine, Southeast University, Nanjing, China; ^4^ Department of Environmental Health, Harvard T.H. Chan School of Public Health, Pulmonary and Critical Care Division, Department of Medicine, Massachusetts General Hospital and Harvard Medical School, Boston, MA, United States

**Keywords:** platelet count, inflammation, immunity, critical care, longitudinal trajectory, multi-cohort, prognosis

## Abstract

**Objective:**

Platelet (PLT) engages in immune and inflammatory responses, all of which are related to the prognosis of critically ill patients. Although thrombocytopenia at ICU admission contributes to in-hospital mortality, PLT is repeatedly measured during ICU hospitalization and the role of longitudinal PLT trajectory remains unclear. We aimed to identify dynamic PLT trajectory patterns and evaluate their relationships with mortality risk and thrombocytopenia.

**Methods:**

We adopted a three-phase, multi-cohort study strategy. Firstly, longitudinal PLT trajectory patterns within the first four ICU days and their associations with 28-day survival were tested in the eICU Collaborative Research Database (eICU-CRD) and independently validated in the Medical Information Mart for Intensive Care IV (MIMIC-IV) database. Secondly, the relationships among PLT trajectory patterns, thrombocytopenia, and 28-day mortality were explored and validated. Finally, a Mortality GRade system for ICU dynamically monitoring patients (Mortality-GRID) was developed to quantify the mortality risk based on longitudinal PLT, which was further validated in the Molecular Epidemiology of Acute Respiratory Distress Syndrome (MEARDS) cohort.

**Results:**

A total of 35,332 ICU patients were included from three cohorts. Trajectory analysis clustered patients into ascending (AS), stable (ST), or descending (DS) PLT patterns. DS patients with high baseline PLT decline quickly, resulting in poor prognosis. AS patients have low baseline PLT but recover quickly, favoring a better prognosis. ST patients maintain low PLT, having a moderate prognosis in between (*HR*_ST_ *_vs_*_AS_ = 1.26, 95% CI: 1.14–1.38, *P* = 6.15 × 10^−6^; *HR*_DS_ *_vs_*_AS_ = 1.58, 95% CI: 1.40–1.79, *P* = 1.41 × 10^−13^). The associations remained significant in patients without thrombocytopenia during the entire ICU hospitalization and were robust in sensitivity analyses and stratification analyses. Further, the trajectory pattern was a warning sign of thrombocytopenia, which mediated 27.2% of the effects of the PLT trajectory on 28-day mortality (*HR*_indirect_ = 1.11, 95% CI: 1.06–1.17, *P* = 9.80 × 10^−6^). Mortality-GRID well predicts mortality risk, which is in high consistency with that directly estimated in MEARDS (*r* = 0.98, *P* = 1.30 × 10^−23^).

**Conclusion:**

Longitudinal PLT trajectory is a complementary predictor to baseline PLT for patient survival, even in patients without risk of thrombocytopenia. Mortality-GRID could identify patients at high mortality risk.

## Introduction

The intensive care unit (ICU) patients are exposed to pneumonia, sepsis, and other inflammation (1, 2), which hampers the treatment and leads to high mortality rates ranging from 11% to 18% (3, 4). Survivors may suffer from chronic and life-changing physical, psychosocial, and cognitive sequelae (5, 6). Such conditions deteriorate rapidly under the ongoing novel coronavirus disease 2019 (COVID-19) pandemic (7) and cause a considerable burden on public health (8, 9).

Platelets engage in coagulation, inflammation, and the immune response, all of which have close connections with the prognosis of critically ill patients (10, 11). Platelets play a central role in regulating immune responses and interactions with various cells of the innate and adaptive immune systems (12, 13). Functional or quantitative platelet abnormalities may lead to immune dysregulation, prolonged ICU stay, and death (14). Indeed, thrombocytopenia (low platelet count) at ICU admission is associated with poor clinical outcomes and prognosis (15, 16). Accumulating pieces of evidence show that platelet counts vary over ICU hospitalization, with levels dropping after ICU admission and reaching a nadir on the fourth day (17, 18). In addition to the baseline platelet count at admission, such platelet dynamics might affect ICU patient prognosis (19). However, the role of the longitudinal platelet count trajectory has not been elucidated.

Taking advantage of large-scale databases including the eICU Collaborative Research Database (eICU-CRD) and the Medical Information Mart for Intensive Care IV (MIMIC-IV) database, we applied an unsupervised machine learning approach to identify longitudinal platelet count trajectory patterns among ICU patients and evaluate their associations with clinical outcomes. Further, we developed a Mortality Grade system for ICU dynamically monitoring patients (Mortality-GRID) and validated it in the Molecular Epidemiology of Acute Respiratory Distress Syndrome (MEARDS) cohort.

## Methods

### Study population

Study samples were collected from eICU-CRD v2.0 (2014–2015) (20) and MIMIC-IV v1.0 (2008–2019) (21) at PhysioNet (22) (certification number: 33755029), as well as the MEARDS cohort (1998–2014) (16, 23). In accordance with previous studies, we excluded ICU admissions if they met any of the following criteria: (i) repeated ICU admissions; (ii) age <18 years; (iii) missing baseline platelet count; (iv) length of ICU stay <48 h (24, 25); (v) daily platelet count measurements <4 times (because of the requirement for trajectory analysis). Detailed cohort descriptions are summarized in the **Supplementary Methods**. Finally, 19,361, 14,239, and 1,732 ICU patients who met the recruitment criteria were collected from the eICU-CRD, MIMIC-IV, and MEARDS cohorts, respectively.

### Clinical outcome and measurements

The primary outcome was 28-day in-hospital survival, defined as a time-to-event outcome from ICU admission to death or loss to follow-up at the end of the study, whichever occurred first. The primary independent variable was daily platelet count measurements in the first four consecutive days after ICU admission. The lowest platelet count was used if the patient had multiple platelet count measures in a single day. Additionally, demographic information, clinical characteristics, laboratory tests, vital signs, and comorbidities were extracted for covariate adjustment and stratified analyses, summarized in **Supplementary Tables 1–5**. Only variables with a missing proportion of less than 10% were included in further analyses (**Supplementary Figures 1, 2**).

### Platelet count dynamic trajectory identification

Trajectory analysis was performed to identify dynamic trends based on longitudinal repeatedly measured platelet count in the first four ICU days using the R package *traj*, as proposed by Leffondré etal. (26). We followed three steps to identify trajectory patterns. Firstly, we extracted 24 features to depict platelet count dynamic trajectories for each individual (**Supplementary Table 6**). Secondly, correlations between these 24 features were tested, and factor analysis was conducted to select a subset of features that best described the dynamic changes of the trajectories. Finally, based on the selected features, cluster analysis was used to classify patients with similar dynamic changes into the same group. The optimal number of clusters was determined based on the lowest −2*log-likelihood value, the Akaike information criterion, and Bayesian information criteria of the corresponding models.

### Causal mediation analysis for dynamic trajectory, thrombocytopenia, and 28-day survival

Thrombocytopenia is an important prognostic predictor of in-hospital mortality in ICU patients. To test whether the dynamic platelet count trajectory could provide warning signs for thrombocytopenia and further affect patient survival, the Vander Weele causal mediation analysis was performed to evaluate the indirect effect of the dynamic trajectory on 28-day survival (27).

### Risk quantization and Mortality-GRID

To quantify the mortality risk of dynamic platelet count changes, we computed the average daily change in platelet count after ICU admission. Considering that patients with different baseline platelet counts at admission may have different mortality risks, we classified patients according to the normal range of platelet counts in the ICU (100–300 × 10^9^/L) (28). Patients with no change in platelet count were set as the reference group, and the effects of platelet count changes on 28-day mortality were evaluated using hazard ratios (HRs) derived from restricted cubic spline using the R package *rms*. Mortality-GRID was developed based on the risk quantization to provide warnings and prophylactic therapy for vulnerable ICU patients.

### Study design and statistical analysis

We conducted a three-phase, multi-cohort study. Firstly, longitudinal platelet count trajectory patterns within the first four ICU days and their associations with 28-day survival were tested in eICU-CRD and further independently validated in MIMIC-IV. Furthermore, a series of stratified and sensitivity analyses were performed to evaluate the robustness of the associations.

Secondly, based on eICU-CRD and MIMIC-IV databases, we explored and validated whether the longitudinal trajectory patterns could provide a warning sign of thrombocytopenia and, through which, mediate the effect of trajectory patterns on patient survival. Additionally, although thrombocytopenia is common in the ICU, considerable patients without thrombocytopenia remain hospitalized during their entire ICU hospitalization. We further clarified the role of dynamic trajectory patterns in such a population.

Finally, we quantified the mortality risk based on the average platelet count change per day and developed Mortality-GRID by combining the eICU-CRD and MIMIC-IV databases to achieve robust parameter estimates. Moreover, the proposed Mortality-GRID was applied in an independent MEARDS cohort. Patients with MEARDS were classified into low-, middle-, and high-risk subgroups by the death hazard tertiles predicted by Mortality-GRID. Then, the survival differences between the subgroups were compared.

The survival differences between trajectory patterns were assessed using multivariable Cox proportional hazards models and illustrated by Kaplan–Meier survival curves. Statistical analyses were performed using the R version 3.6.3. The significance level was defined as two-sided 0.05. The source code was deposited on GitHub: https://github.com/JiajinChen/trajPLT.

## Results

### Platelet count trajectories and patient characteristics

**Figure 1:**
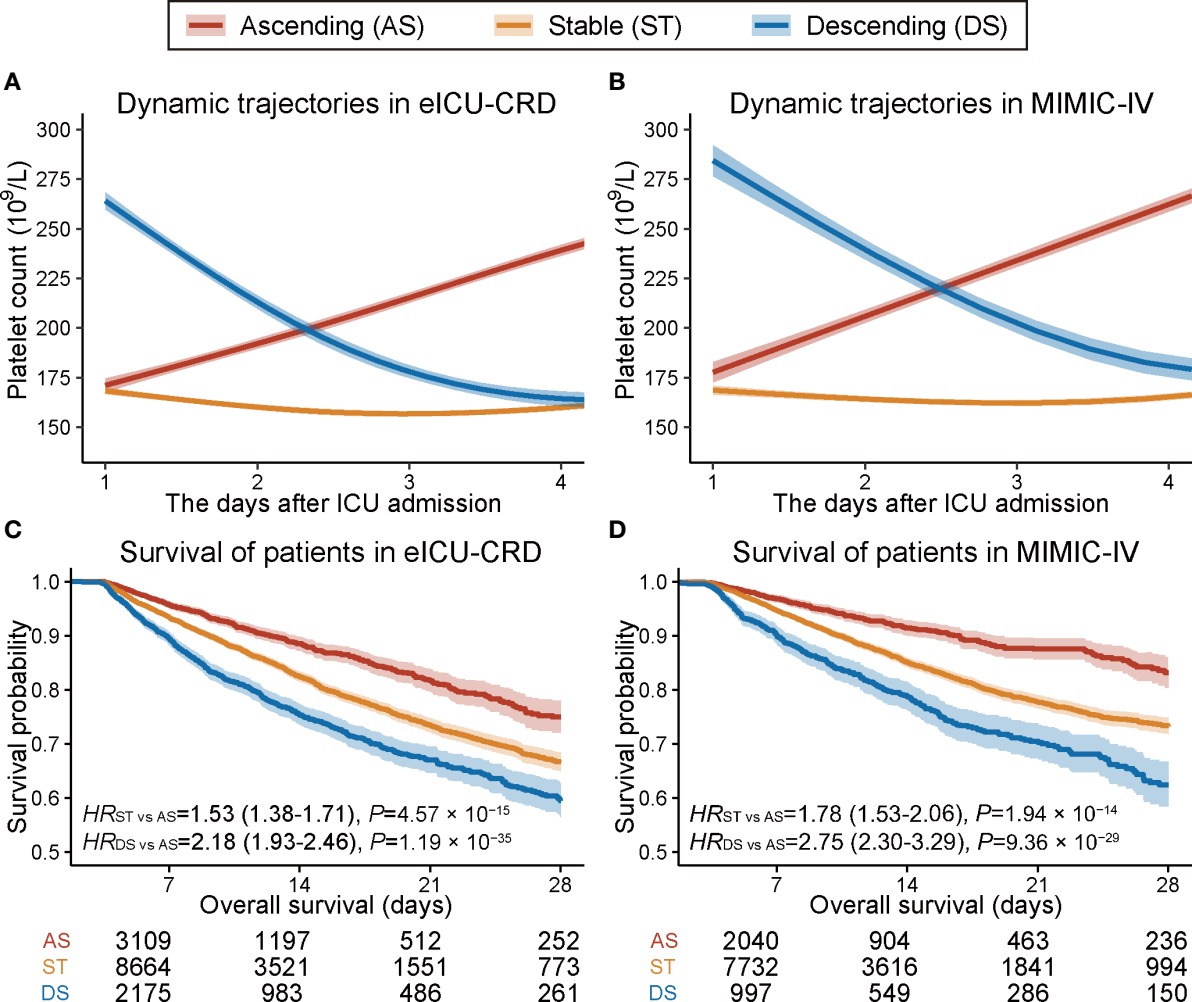
Trajectory plot and Kaplan–Meier survival curves of patients with three dynamic platelet count trajectory patterns. **(A, B)** Trajectory plot of platelet count changes within the first four days after ICU admission in eICU-CRD and MIMIC-IV databases. **(C, D)** Kaplan–Meier curves of 28-day overall survival for patients with three different dynamic platelet count trajectory patterns in the eICU-CRD and MIMIC-IV databases.

Trajectory analysis identified three distinct longitudinal platelet count trajectory patterns in eICU-CRD (**Supplementary Table 7**): (i) ascending (AS), in which the platelet count was elevated after ICU admission; (ii) stable (ST), in which the platelet count remained relatively unchanged; and (iii) descending (DS), in which the platelet count declined quickly after ICU admission (**Figure 1A**). Similar patterns were observed upon validation in the MIMIC-IV dataset using the model trained in eICU-CRD (**Figure 1B**).

A broad list of demographics, clinical characteristics, laboratory measurements, and vital signs collected at ICU admission were compared across the three trajectory patterns in eICU-CRD (**Supplementary Tables 8–11**) and MIMIC-IV databases (**Supplementary Tables 12–15**). Significantly different characteristics across these three patterns were considered potential covariates and adjusted in subsequent models.

### Platelet count trajectory patterns and 28-day survival

A univariate Cox proportional hazards model indicated that patients with ST or DS patterns had significantly worse 28-day survival than those with an AS pattern in eICU-CRD (**Figure 1C**) and MIMIC-IV datasets (**Figure 1D**). Further, we verified the robustness of the results by developing six models adjusted for a list of sequentially adding-on covariates (Model_1–5_ and Model_PS_ in **Table 1**). The model was adjusted first for demographics and baseline platelet count (Model_1_), followed by step-forward add-on adjustments for predispositions and supports (Model_2_), vital signs (Model_3_), unevenly distributed laboratory measurements (Model_4_), and comorbidities (Model_5_). All models retained consistently significant associations (**Table 1**). Further, the propensity score model incorporating all these covariates retained consistent results (*HR*_ST_ *_vs_*_AS_ = 1.26, 95% CI: 1.14–1.38, *P* = 6.15 × 10^−6^; *HR*_DS_ *_vs_*_AS_ = 1.58, 95% CI: 1.40–1.79, *P* = 1.41 × 10^−13^) (Model_PS_ in **Table 1**). Moreover, the associations remained significant in subgroup analyses (**Supplementary Figure 3**).

**Table 1:** Sensitivity analyses for association between platelet count trajectories and 28-day overall survival.

Model	Cluster	eICU-CRD			MIMIC-IV			Meta		
		*HR*	95% CI	*P*	*HR*	95% CI	*P*	*HR*	95% CI	*P*
Model_1_	Ascending	Reference			Reference			Reference		
	Stable	1.44	(1.30–1.61)	1.85 × 10^−11^	1.61	(1.39–1.87)	2.25 × 10^−10^	1.50	(1.37–1.63)	8.89 × 10^−20^
	Descending	2.33	(2.05–2.65)	4.94 × 10^−38^	2.81	(2.33–3.38)	2.01 × 10^−27^	2.48	(2.23–2.75)	1.93 × 10^−63^
Model_2_	Ascending	Reference			Reference			Reference		
	Stable	1.29	(1.15–1.44)	9.64 × 10^−6^	1.58	(1.36–1.83)	1.36 × 10^−9^	1.39	(1.27–1.52)	6.68 × 10^−13^
	Descending	1.67	(1.45–1.91)	2.26 × 10^−13^	2.00	(1.65–2.42)	1.12 × 10^−12^	1.78	(1.59–1.99)	7.86 × 10^−24^
Model_3_	Ascending	Reference			Reference			Reference		
	Stable	1.28	(1.14–1.43)	4.17 × 10^−5^	1.45	(1.25–1.69)	1.17 × 10^−6^	1.35	(1.23–1.48)	1.22 × 10^−10^
	Descending	1.61	(1.39–1.86)	8.23 × 10^−11^	1.78	(1.47–2.17)	7.59 × 10^−9^	1.67	(1.49–1.88)	7.41 × 10^−18^
Model_4_	Ascending	Reference			Reference			Reference		
	Stable	1.28	(1.13–1.45)	1.10 × 10^−4^	1.33	(1.14-1.56)	3.35 × 10^−4^	1.30	(1.18–1.43)	1.47 × 10^−7^
	Descending	1.66	(1.42–1.94)	1.07 × 10^−10^	1.72	(1.40–2.12)	2.20 × 10^−7^	1.68	(1.48–1.90)	3.14 × 10^−16^
Model_5_	Ascending	Reference			Reference			Reference		
	Stable	1.24	(1.10–1.41)	6.03 × 10^−4^	1.28	(1.10–1.50)	2.01 × 10^−3^	1.26	(1.14–1.38)	6.15 × 10^−6^
	Descending	1.61	(1.38–1.88)	1.25 × 10^−9^	1.58	(1.29–1.94)	1.35 × 10^−5^	1.60	(1.41–1.81)	8.26 × 10^−14^
Model_PS_	Ascending	Reference			Reference			Reference		
	Stable	1.24	(1.09–1.40)	8.95 × 10^−4^	1.28	(1.09–1.50)	2.01 × 10^−3^	1.26	(1.14–1.38)	6.15 × 10^−6^
	Descending	1.59	(1.36–1.84)	3.36 × 10^−9^	1.57	(1.28–1.93)	1.54 × 10^−5^	1.58	(1.40–1.79)	1.41 × 10^−13^

Model_1_: adjusted for age, gender, ethnicity, baseline platelet count, antiplatelet treatment, platelet transfusion, transfusion amount, malignancies, hematologic diseases, immune therapy, thrombotic diseases, and thromboinflammatory diseases.

Model_2_: additionally adjusted for first ICU location, ARDS, sepsis, SOFA, APS-III, and supports within 24 h (mechanical ventilation, vasopressor, and dialysis) upon model_1_.

Model_3_: additionally adjusted for differential vital signs upon model_2_.

Model_4_: additionally adjusted for differential laboratory results upon model_3_.

Model_5_: additionally adjusted for differential comorbidities upon model_4_.

Model_PS_: adjusted for all aforementioned covariates using the propensity score (PS) method.

### Stratified analyses for associations between trajectory patterns and 28-day survival

**Figure 2:**
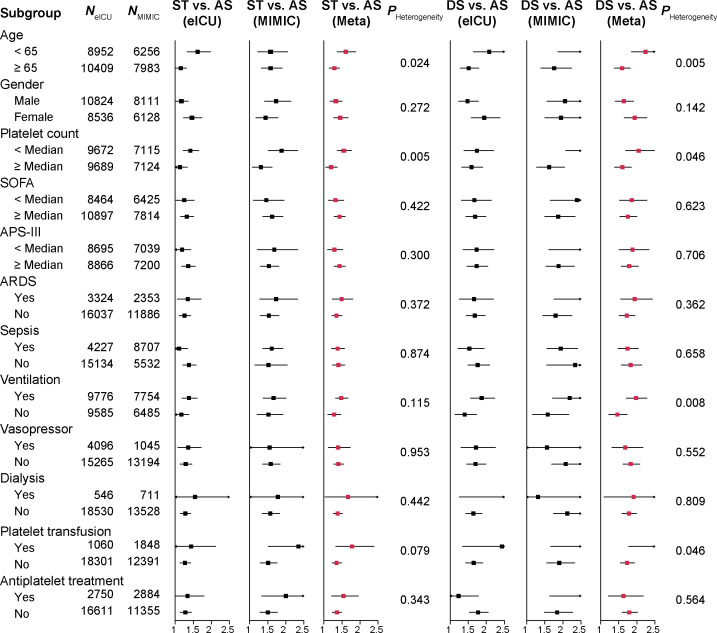
Forest plots of stratified associations between platelet count trajectory patterns and 28-day survival of ICU patients. Meta-analysis was conducted to pool the results from the eICU-CRD and MIMIC-IV databases. The effects across strata were tested using heterogeneity test.

Furthermore, a series of analyses stratified by demographics, severity score, support within 24 h, and comorbidities were performed. Significant associations between platelet count dynamic trajectory pattern and 28-day mortality were observed in most strata in the eICU-CRD and MIMIC-IV databases (**Figure 2** and **Supplementary Figures 4–5**), except for a few strata probably due to insufficient sample size (**Supplementary Figures 6–11**). Notably, significant heterogeneity in the association was observed between the age subgroups. ICU patients aged <65 years with either an ST (*P*_Heterogeneity_ = 0.024) or DS (*P*_Heterogeneity_ = 0.005) dynamic platelet count trend had higher mortality than patients ≥65 years old (**Figure 2**). Also, significant heterogeneity was observed in the different baseline platelet count subgroups at ICU admission (*P*_Heterogeneity_ *<*0.05) (**Figure 2**).

### Relationship between platelet count trajectory, thrombocytopenia, and 28-day survival

Thrombocytopenia is an important prognostic predictor of ICU patient mortality, regardless of when it occurs (**Supplementary Table 16**). Furthermore, we observed the dynamic platelet count trajectory during the first four ICU days as a powerful predictor of thrombocytopenia risk during the following days of ICU hospitalization in the eICU-CRD (*OR*_ST_ *_vs_*_AS_ = 3.20, 95% CI: 2.36–4.33, *P* = 5.73 × 10^−14^; *OR*_DS_ *_vs_*_AS_ = 6.84, 95% CI: 4.95–9.45, *P* = 1.73 × 10^−31^) and the MIMIC-IV dataset (*OR*_ST_ *_vs_*_AS_ = 2.58, 95% CI: 1.84–3.61, *P* = 3.47 × 10^−8^; *OR*_DS_ *_vs_*_AS_ = 6.27, 95% CI: 4.27–9.20, *P* = 7.61 × 10^−21^). The associations were robust in six models, adjusting for a list of sequentially adding-on covariates (**Supplementary Table 17**).

**Figure 3:**
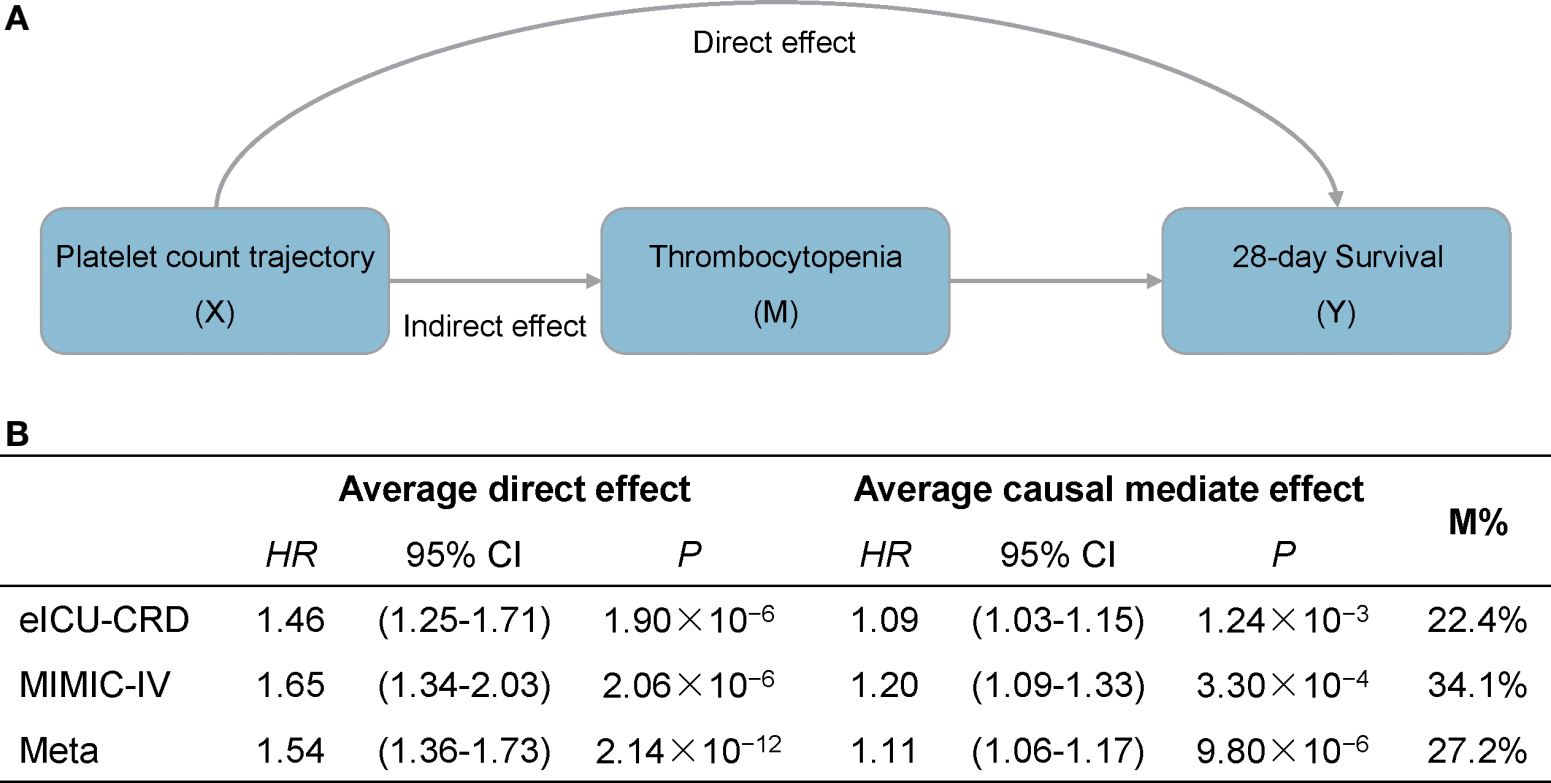
Causal mediation analysis for platelet count dynamic trajectory, thrombocytopenia, and 28-day survival. **(A)** Mediation model for the effect of platelet count dynamic trajectory on 28-day survival through thrombocytopenia. **(B)** Results are described as average causal mediated effect (indirect hazard ratio), 95% confidence interval and the proportion of effect mediated (M%).

Furthermore, to test whether dynamic platelet count trajectory in the first four days after ICU admission could affect patient survival *via* thrombocytopenia, we performed a causal mediation analysis and a significant indirect effect on 28-day survival was observed in both eICU-CRD and MIMIC-IV datasets (*HR*_Indirect_ = 1.11, 95% CI: 1.06–1.17, *P* = 9.80 × 10^−6^, 27.2% of effects mediated) (**Figure 3**). On the other hand, there were still over 70% of the effects of platelet count trajectory affecting patient mortality through underlying mechanisms rather than thrombocytopenia.

### Trajectory patterns and 28-day mortality in patients without thrombocytopenia

Indeed, though thrombocytopenia is common in critically ill patients (31.2% in eICU-CRD and 30.1% in MIMIC-IV), a considerable proportion of patients without thrombocytopenia remain during the entire course of ICU hospitalization. Hence, we further conducted a subgroup analysis among patients with no history of thrombocytopenia during the whole ICU hospitalization. The associations between platelet count dynamic trajectory and 28-day mortality remained significant in eICU-CRD (*HR*_ST_ *_vs_*_AS_ = 1.22, 95% CI: 1.02–1.47, *P* = 3.02 × 10^−2^; *HR*_DS_ *_vs_*_AS_ = 1.73, 95% CI: 1.39–2.15, *P* = 9.41 × 10^−7^) and MIMIC-IV (*HR*_ST_ *_vs_*_AS_ = 1.48, 95% CI: 1.17–1.87, *P* = 9.11 × 10^−4^; *HR*_DS_ *_vs_*_AS_ = 2.13, 95% CI: 1.57–2.88, *P* = 1.13 × 10^−6^), respectively (**Supplementary Figure 12**), suggesting that dynamic platelet count trajectory was an independent prognostic predictor of 28-day mortality of ICU patients, even in patients who never developed thrombocytopenia.

### Dynamic platelet count change and mortality quantization

Considering the heterogeneity between patients with different baseline platelet counts at ICU admission, we stratified patients according to the normal platelet count range. A significant dose–response relationship between daily platelet count change and 28-day mortality was observed in the eICU-CRD and MIMIC-IV datasets (**Supplementary Figure 13**). Compared to patients with no change in platelet count, patients with a daily decline in platelet count in the first four days by 10 × 10^9^/L and a baseline platelet count of <100, between 100 and 300, or >300 had a 51% (*HR* = 1.51, 95% CI: 1.25–1.83), 16% (*HR* = 1.16, 95% CI: 1.12–1.20), or 8% (*HR* = 1.08, 95% CI: 1.03–1.13) increased risk of death, respectively. On the other hand, patients with a baseline platelet count below 100 × 10^9^/L and a daily platelet count increment of 10 × 10^9^/L, a baseline platelet count in the normal range with a daily increment reaching 22 × 10^9^/L, or a baseline platelet count more than 300 × 10^9^/L with an increment of 31 × 10^9^/L, had a 25% decreased risk of death (**Supplementary Figure 13**).

### Development and validation of the Mortality-GRID

**Figure 4:**
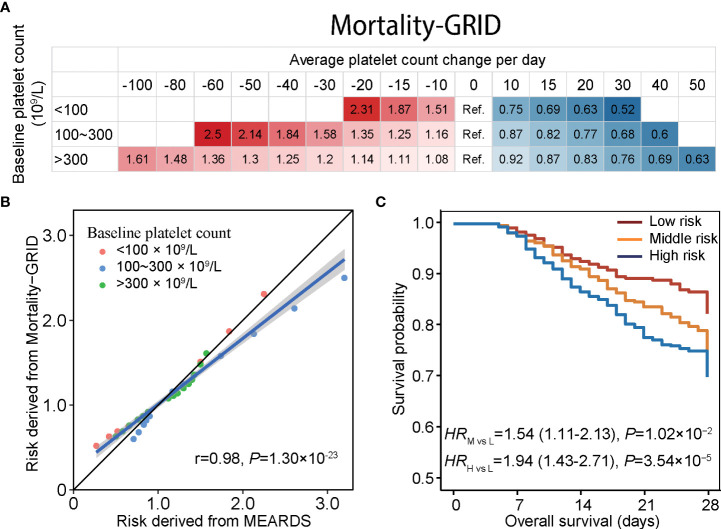
Mortality-GRID and validation in the MEARDS database. **(A)** Mortality-GRID. Patients with no change in platelet count per day were set as the reference group, the values in each cell represent hazard ratios of platelet count changes per day derived from restricted cubic spline regression. **(B)** Consistency between mortality risks estimated by Mortality-GRID and MEARDS database presented by scatter plot. **(C)** Estimated survival curves for patients in the MEARDS database. The mortality risks of MEARDS patients were independently predicted by Mortality-GRID and categorized into low-, medium-, and high-risk groups using the tertiles.

Based on the mortality quantization, we derived Mortality-GRID using the baseline platelet count at ICU admission and daily platelet count change in the first four days of ICU hospitalization to indicate patient death hazards (**Figure 4A**). Using this model, one can assign a hazard risk to a patient by their average daily platelet count change. For example, suppose ICU patients with a baseline platelet count of 200 × 10^9^/L have an average platelet count decline of 30 × 10^9^/L, which might cause a 1.58-times mortality risk compared to those with an average platelet count change equal to 0 (stable status). Subsequently, we independently estimated the corresponding risk of daily platelet count change across different baseline platelet count groups in MEARDS (**Supplementary Figure 14**). Mortality risks predicted by Mortality-GRID were consistent with those directly estimated in MEARDS (*r* = 0.98, *P* = 1.30 × 10^−23^) (**Figure 4B**), indicating the clinical applicability of Mortality-GRID. Furthermore, we applied Mortality-GRID to the MEARDS cohort and predicted patients’ hazard risk. Patients were then categorized into high-, middle-, and low-risk groups using the tertiles of their hazard risk values. Based on the significant survival differences between groups, the proposed Mortality-GRID successfully identified patients with unfavorable survival (**Figure 4C**).

## Discussion

To our knowledge, this is the first study to integrate multiple large-scale databases and apply machine learning methods to identify longitudinal platelet count trajectory patterns and validate their relationship with the prognosis of ICU patients. Such associations were robust in sensitivity analyses, and even remained significant among patients without thrombocytopenia during the entire ICU hospitalization. Moreover, the trajectory is a warning sign of thrombocytopenia, which affects patient survival.

Compared to the absolute platelet count at a specific time, longitudinal data provide more information on disease progression (29, 30). Trajectory analysis can uncover the hidden values of repeated measurements. Our study indicated that patients without thrombocytopenia at ICU admission but who had a rapidly declining platelet count during hospitalization might eventually have a poor prognosis.

Among the three trajectory patterns, the descending pattern was characterized as having a high mortality risk, whereas ICU patients with an elevated platelet count had a favorable clinical outcome. Platelets are well recognized in hemostasis by promoting vasoconstriction and platelet aggregation. Moreover, platelets can release proangiogenic factors (such as VEGF) that induce the proliferation and migration of fibroblasts and endothelial cells, thereby contributing to the repair of vascular epithelial damage (31). Most ICU patients experience inflammatory stress or infection, while platelets induce the proliferation, maturation, and activation of immune cells and interact with Toll-like receptors (TLRs) (32), which can recognize pathogen or virus, and are involved in the immune response (33). Thrombocytopenia is a common sign of poor prognosis in ICU patients (34). The decline of platelet count may result in weakened immune function, while the increased platelet count may reflect the elimination of pathogens and the recovery of immune defense (35–37), which contributes to the recovery of ICU patients. Nevertheless, excess platelets are a risk factor for thromboembolism (38), and special attention is required for ICU patients at risk of thromboembolic diseases.

A series of stratified analyses showed robust associations across strata. Notably, we found the risk of descending platelet count was lower among elderly adults (≥65 years). In older adults, platelet activation, aggregation, and secretion were enhanced, and maintaining a relatively low platelet count may relieve the risk of thrombosis and inflammation (39, 40). Further platelet count decline may exacerbate the incidence of adverse events among patients with a low platelet count at admission (41). Moreover, platelet engage in the antivirus and anti-infection processes by forming neutrophil extracellular traps (NET), which enhance leukocyte recruitment and inflammatory factor activation (42). These mechanisms might induce plasma coagulation factors, resulting in pro-inflammatory responses and thrombosis (43). Our results also indicated the heterogeneous effects of platelet trajectory between patients with and without thromboembolic diseases, suggesting the necessity of maintaining a relatively balanced platelet count in patients with thromboembolic disease. Although the associations between platelet trajectory and 28-day mortality were not observed in the subgroup of patients with thromboinflammatory diseases, that might be due to the insufficient sample size. The further heterogeneity suggested no heterogeneity between patients with and without thromboinflammatory diseases. Nevertheless, emerging pieces of evidence have underlined the unique role of platelets in COVID-19, a thromboinflammatory disease (44, 45). During COVID-19, platelets get activated through multiple mechanisms involving the global inflammatory reaction, the dysfunctional endothelium, increased thrombin, or plausible direct viral infection (44). Moreover, the activated platelets can interact with neutrophils, which mediated the inflammation and thrombosis (46). The platelet–neutrophil interaction may trigger the release of NETs (47), which contribute to acute organ failure, ARDS, and mortality in COVID-19 (48). Further data is needed for validation of our model in populations with COVID-19. In addition, several factors (i.e., platelet treatment, transfusion amount, hematologic diseases) may affect the associations. We therefore adjusted them in the models and performed sensitivity analyses by excluding patients with platelet treatment. The associations remained significant in the sensitivity analyses, indicating the robustness of our results.

Additionally, our results showed that the dynamic trajectory of platelet count could be a warning sign for thrombocytopenia and affect ICU patient mortality. For about 30% of the effect of platelet count decline in mortality that is mediated by thrombocytopenia, the trajectory patterns could provide considerable information through other underlying mechanisms. Platelet count can reflect the function and stress response of organs, such as the lung, liver, and kidney (49, 50). Notably, even in patients without thrombocytopenia during their entire ICU hospitalization, the dynamic change in platelet count retained a significant effect on prognosis, which might benefit nearly 70% of ICU patients without thrombocytopenia. Although the daily platelet change seems small, it reflects the dynamic trend, which powerfully predicts mortality. Consequently, the Mortality-GRID system based on accessible daily platelet count could help identify high-risk patients, aid clinical decisions, and guide therapy.

Our study has several strengths. First, we analyzed two large-scale databases, providing robust results. Moreover, these two databases have extensive documentation and public code for researchers. We also released the source code for the audit and tried to ensure reliable data quality and results. Second, we used longitudinal platelet count measures over the first four days of ICU hospitalization to identify three novel dynamic trajectory patterns, which could predict patient prognosis and would considerably benefit clinical decision-making. Third, we used the causal inference method and confirmed the applicability of trajectory patterns for warning signs in patients without thrombocytopenia. Fourth, we adopted a three-phase study design and unsupervised trajectory analysis to better identify the underlying information, along with a series of sensitivity analyses and stratified analyses to guarantee the robustness of the findings. Finally, we provided Mortality-GRID to assist physicians with warning sign detection based on platelet count changes, dynamical assessment for mortality hazards, and early identification of high-risk patients, and then provide personalized prophylactic therapy, i.e., platelet therapy.

We acknowledge some limitations in our study. First, since our samples are mainly Caucasian, a large dataset with other ethnicities is warranted to confirm the applicability of our findings across different ethnicities. Second, more patients had sepsis predisposition in MIMIC-IV than in eICU-CRD. The database constructor provided sepsis 3.0 diagnostics in MIMIC-IV; this variable was not provided in the eICU-CRD database and was manually extracted from the diagnostic texts; thus, sepsis was probably under-diagnosed. However, no heterogeneity was observed between sepsis and non-sepsis subgroups in the two databases. Thus, the potential under-diagnosis of sepsis in eICU-CRD had no impact on the findings of this study. Third, although our study included three databases, heterogeneity might exist across these databases. Nevertheless, the association remained significant across these heterogeneous populations, indicating the robustness of our results.

In summary, we identified three distinct longitudinal platelet count trajectory patterns within the first four ICU days. The platelet count trajectory is a better predictor of patient survival than merely the baseline platelet count. These associations are partially mediated by thrombocytopenia and remain significant in patients without risk of thrombocytopenia. The proposed Mortality-GRID could facilitate prognostic warnings for vulnerable ICU patients.

## Data availability statement

The MIMIC-IV and eICU-CRD databases are available in PhysioNet (https://physionet.org). MEARDS cohort is available from the corresponding authors on reasonable request.

## Ethics statement

eICU and MIMIC databases were approved by the Institutional Review Boards (IRB) of the Massachusetts Institute of Technology, and the informed consent has been waived as they do not contain protected health information. MEARDS cohort was reviewed and approved by IRB of Harvard T. H. Chan School of Public Health, MGH and BIDMC, and all participants provided their written informed consent.

## Author contributions

YW and RZ designed and supervised the study. JC and LS curated and harmonized the data. JC, XG, SS, and ZS performed statistical analyses. ZD and RL double-checked all analysis code. JC, XG, and LS drafted the manuscript. FC and YW critically reviewed all statistical methods, procedures, and results. DC provided data from MEARDS and reviewed all clinical interpretations. SS, RZ, YW, FC, and DC critically revised the manuscript. All authors listed have made a substantial, direct, and intellectual contribution to the work and approved it for publication.

## Funding

This study was supported by the National Natural Science Foundation of China (81973142 to YW), the Natural Science Foundation of Jiangsu Province (BK20191354 to RZ), the China Postdoctoral Science Foundation (2020M681671 to SS), and the Jiangsu Planned Projects for Postdoctoral Research Funds (2020Z019 to SS). RZ was partially supported by the Qing Lan Project of the Higher Education Institutions of Jiangsu Province and the Outstanding Young Level Academic Leadership Training Program of Nanjing Medical University.

## Acknowledgments

The authors acknowledge all participants in the eICU, MIMIC, and MEARDS studies, and the Laboratory for Computational Physiology team from the Massachusetts Institute of Technology (LCP-MIT) who maintain the eICU-CRD and MIMIC-IV databases.

## Conflict of interest

The authors declare that the research was conducted in the absence of any commercial or financial relationships that could be construed as a potential conflict of interest.

## Publisher’s note

All claims expressed in this article are solely those of the authors and do not necessarily represent those of their affiliated organizations, or those of the publisher, the editors and the reviewers. Any product that may be evaluated in this article, or claim that may be made by its manufacturer, is not guaranteed or endorsed by the publisher.

## Abbreviations

eICU-CRD, eICU Collaborative Research Database; MIMIC-IV, Medical Information Mart for Intensive Care IV database; ICU, intensive care unit; MEARDS, Molecular Epidemiology of Acute Respiratory Distress Syndrome; PLT, platelet count; Mortality-GRID, Mortality Grade system for ICU Dynamically monitoring patients; CI, confidence interval; HR, hazard ratios; OR, odds ratio; PS, propensity score.
