# Supplementary material for: Association of longitudinal platelet count trajectory with ICU mortality: A multi-cohort study
Source: Front Immunol. 2022 Aug 19;13:936662. doi: 10.3389/fimmu.2022.936662 (PMC9437551; doi:10.3389/fimmu.2022.936662)
Supplement: Supplementary file 1 [file DataSheet_1.docx]

**Online Supplements**

Association of Longitudinal Platelet Count Trajectory with ICU Mortality: A Multi-cohort Study

Jiajin Chen^1†^, Xi Gao^2†^, Sipeng Shen^1†^, Jingyuan Xu^3^, Zhe Sun^1^, Ruilang Lin^1^, Zhixiang Dai^1^, Li Su^4^, David C. Christiani^4^, Feng Chen^1^, Ruyang Zhang^1*^, Yongyue Wei^1*^

**Affiliations:**

^1^ Department of Biostatistics, Center for Global Health, School of Public Health, Nanjing Medical University, Nanjing, Jiangsu 211166, China

^2^ Department of Immunology, School of Clinical Medicine, Nanjing Medical University, Nanjing, Jiangsu 211166, China

^3^ Department of Critical Care Medicine, Zhongda Hospital, School of Medicine, Southeast University, Nanjing, Jiangsu 210009, China.

^4^ Department of Environmental Health, Harvard T.H. Chan School of Public Health, Pulmonary and Critical Care Division, Department of Medicine, Massachusetts General Hospital and Harvard Medical School, Boston, MA 02114, USA

# **Supplementary Methods**

**Cohort descriptions and study patients**

***eICU-CRD***

The Philips eICU program is a transformational critical care telehealth program that provides 24-hour support for caregivers at the bedside. The eICU-CRD v2.0 encompasses more than 200,859 ICU admissions of 139,367 unique patients from 208 hospitals across the United States in 2014 and 2015 (Pollard et al., 2018). The database includes vital sign measurements, care plan documentation, severity of illness measures, diagnosis information, and treatment information. All tables are de-identified to meet the safe harbor provision of the US Health Insurance Portability and Accountability Act (HIPAA). These provisions include removal of all protected health information. We used the eICU-code repository to define concepts in eICU 2.0. After excluding 84,930 admissions that were repeated or cannot be defined due to lack of admission time, 115,929 first ICU admissions were retained. Among which, 96,568 were excluded for the following reasons: age < 18 years (479); missing baseline platelet count (6,988); length of ICU stays < 48 hours (65,997); daily platelet count < 4 times (23,104), 19,361 ICU admissions were included in further study.

***MIMIC-IV***

The Medical Information Mart for Intensive Care (MIMIC) database is retrospectively collected from digital electronic health records, which provide critical care data to improve patient care through knowledge discovery and algorithm development. The MIMIC-IV v1.0 covers 76,540 ICU admissions of 53,150 unique patients between 2008 and 2019 from the Beth Israel Deaconess Medical Center (BIDMC) in Boston, Massachusetts (Johnson et al., 2016). The database was prepared in three steps: acquisition, preparation, and deidentification and further grouped into Core, Hosp, and ICU modules. We extracted the data according to the MIMIC Code Repository. A total of 76,540 ICU admissions were extracted and 53,150 were retained after excluded repeated admissions. 1,240 were excluded for missing baseline platelet count; 27,326 for length of ICU stays < 48 hours; and 10,345 for daily platelet count < 4 times, 14,239 eligible ICU admissions were retained in the sequent study.

The eICU-CRD and MIMIC-IV databases are hosted by the Laboratory for Computational Physiology at the Massachusetts Institute of Technology (Goldberger et al., 2000). One author (J.C.) obtained access and was responsible for data extraction (certification number 33755029). Detailed demographic information, clinical characteristics, laboratory tests, vital signs, and comorbidities were extracted accordingly (Serpa Neto et al., 2018;van den Boom et al., 2020).

***MEARDS***

The MEARDS prospective cohort study (ClinicalTrials.gov Identifier: NCT00006496) contains 6,679 critically-ill patients, who were recruited from the ICUs of Massachusetts General Hospital (MGH) and the BIDMC between 1998 and 2014 (Wei et al., 2017;Zhang et al., 2017). The study was reviewed and approved by institutional review boards of Harvard T. H. Chan School of Public Health, MGH and BIDMC. All participants or their surrogate care providers gave written informed consent. Of the 6,679 ICU admissions, we excluded 620 repeated admissions, 664 with missing baseline platelet counts, 1296 with length of stays < 48 hours, and 2367 with daily platelet count < 4 times. Finally, 1,732 ICU admissions met the criteria were included as analytical set.

**Statistical analysis**

Continuous variables that followed a normal distribution were summarized as mean and standard deviation (SD), and were compared using Student’s t-test or analysis of variance (ANOVA) between different platelet count dynamic trajectory patterns. Non-normal distributed continuous variables were described as median and interquartile range (IQR), and were compared using the Wilcox rank-sum test or Kruskal-Wallis test across patterns. Categorical variables were described by frequency (n) and proportion (%), and were tested using a Chi-squared test or Fisher's exact test. Variables significantly different across patterns were considered potential covariates and were adjusted in the subsequent association analysis using the Cox proportional hazards model.

The association between dynamic platelet count trajectory pattern and 28-day overall survival was tested by the Cox proportional hazards model adjusted for covariates. Kaplan-Meier survival curves were used to illustrate survival differences between patients with different patterns. Logistic regression models were performed to evaluate the associations between platelet count trajectory patterns and risk of thrombocytopenia adjusted for covariates. The indirect effects of platelet count dynamic trajectory on survival via thrombocytopenia development were evaluated by causal mediation analysis, and Monte Carlo bootstrapping resampling 1,000 times generated the standard errors (VanderWeele, 2011). Sensitivity analyses with adjustment for gradually added on covariates, and propensity score analyses incorporating all covariates were performed. The effects across strata were tested using heterogeneity test. The association results from eICU-CRD and MIMIC-IV datasets were then pooled by meta-analysis using R package *meta*.

**Reference**

Goldberger, A.L., Amaral, L.A., Glass, L., Hausdorff, J.M., Ivanov, P.C., Mark, R.G., Mietus, J.E., Moody, G.B., Peng, C.-K., and Stanley, H.E. (2000). PhysioBank, PhysioToolkit, and PhysioNet: components of a new research resource for complex physiologic signals. *Circulation* 101**,** e215-e220.

Johnson, A.E., Pollard, T.J., Shen, L., Lehman, L.W., Feng, M., Ghassemi, M., Moody, B., Szolovits, P., Celi, L.A., and Mark, R.G. (2016). MIMIC-III, a freely accessible critical care database. *Sci Data* 3**,** 160035.

Pollard, T.J., Johnson, A.E.W., Raffa, J.D., Celi, L.A., Mark, R.G., and Badawi, O. (2018). The eICU Collaborative Research Database, a freely available multi-center database for critical care research. *Sci Data* 5**,** 180178.

Serpa Neto, A., Deliberato, R.O., Johnson, A.E.W., Bos, L.D., Amorim, P., Pereira, S.M., Cazati, D.C., Cordioli, R.L., Correa, T.D., Pollard, T.J., Schettino, G.P.P., Timenetsky, K.T., Celi, L.A., Pelosi, P., Gama De Abreu, M., and Schultz, M.J. (2018). Mechanical power of ventilation is associated with mortality in critically ill patients: an analysis of patients in two observational cohorts. *Intensive Care Med* 44**,** 1914-1922.

Van Den Boom, W., Hoy, M., Sankaran, J., Liu, M., Chahed, H., Feng, M., and See, K.C. (2020). The Search for Optimal Oxygen Saturation Targets in Critically Ill Patients: Observational Data From Large ICU Databases. *Chest* 157**,** 566-573.

Vanderweele, T.J. (2011). Causal mediation analysis with survival data. *Epidemiology* 22**,** 582-585.

Wei, Y., Tejera, P., Wang, Z., Zhang, R., Chen, F., Su, L., Lin, X., Bajwa, E.K., Thompson, B.T., and Christiani, D.C. (2017). A Missense Genetic Variant in LRRC16A/CARMIL1 Improves Acute Respiratory Distress Syndrome Survival by Attenuating Platelet Count Decline. *Am J Respir Crit Care Med* 195**,** 1353-1361.

Zhang, R., Wang, Z., Tejera, P., Frank, A.J., Wei, Y., Su, L., Zhu, Z., Guo, Y., Chen, F., Bajwa, E.K., Thompson, B.T., and Christiani, D.C. (2017). Late-onset moderate to severe acute respiratory distress syndrome is associated with shorter survival and higher mortality: a two-stage association study. *Intensive Care Med* 43**,** 399-407.

# **Supplementary Table 1.** Description of demographics, severity scores, and treatment supports for eligible patients in the eICU-CRD, MIMIC-IV and MEARDS databases

| **Variable** | **eICU-CRD**  **(*n* = 19,361)** | **MIMIC-IV**  **(*n* = 14,239)** | **MEARDS**  **(*n* = 1,732)** |
| --- | --- | --- | --- |
| Age (years) | 66.00 (54.00-77.00) | 67.61 (55.68-78.34) | 63.00 (49.00-74.00) |
| Gender, male | 10824 (55.91%) | 8111 (56.96%) | 1049 (60.57%) |
| Ethnicity |  |  |  |
| Caucasian | 14753 (76.20%) | 9336 (65.57%) | 1564 (90.30%) |
| African American | 2164 (11.18%) | 1177 (8.27%) | 59 (3.41%) |
| Asian | 348 (1.80%) | 378 (2.65%) | 25 (1.44%) |
| Hispanic | 753 (3.89%) | 451 (3.17%) | 75 (4.33%) |
| Native American | 107 (0.55%) | 31 (0.22%) | 1 (0.06%) |
| Other/Unknown | 1236 (6.38%) | 2866 (20.13%) | 8 (0.46%) |
| First ICU location |  |  |  |
| Cardiac ICU | 4286 (22.14%) | 4267 (29.97%) | 269 (15.53%) |
| Med-Surg ICU | 10172 (52.54%) | 1989 (13.97%) | 2 (0.12%) |
| MICU | 1705 (8.81%) | 2561 (17.99%) | 876 (50.58%) |
| Neuro ICU | 1592 (8.22%) | 1109 (7.79%) | 63 (3.64%) |
| SICU | 1606 (8.30%) | 4313 (30.29%) | 522 (30.14%) |
| ARDS at baseline | 3324 (17.17%) | 2353 (16.53%) | 345 (19.92%) |
| Mild | 1030 (5.32%) | 695 (4.88%) | 7 (0.40%) |
| Moderate | 1447 (7.47%) | 1058 (7.43%) | 172 (9.93%) |
| Severe | 847 (4.38%) | 600 (4.21%) | 166 (9.58%) |
| Sepsis at baseline | 4227 (21.83%) | 8707 (61.15%) | 1614 (93.19%) |
| Immunotherapy | 93 (0.48%) | 175 (1.23%) | - |
| Hematologic diseases^1^ | 953 (4.92%) | 306 (2.15%) | - |
| Thromboinflammatory diseases^2^ | 767 (3.96%) | 837 (5.88%) | - |
| Thrombotic diseases^3^ | 3513 (18.14%) | 4523 (31.76%) | - |
| Severity of illness |  |  |  |
| SOFA | 6.00 (4.00-8.00) | 6.00 (4.00-9.00) | - |
| APS-III | 51.00 (36.00-70.00) | 52.00 (37.00-73.00) | 59.00 (46.00-73.00) |
| Platelet treatment |  |  |  |
| Platelet transfusion | 1060 (5.47%) | 1848 (12.98%) | 127 (7.33%) |
| Platelet amount^4^ | 443.5 (195.0-1819.63) | 435.5 (192.0-2869.0) | - |
| Antiplatelet treatment | 2750 (14.20%) | 2884 (20.25%) | - |
| Support within the first 24 h |  |  |  |
| Mechanical ventilation | 9776 (50.49%) | 7754 (54.46%) | 1356 (78.29%) |
| Vasopressor | 4096 (21.16%) | 1045 (7.34%) | 1482 (85.57%) |
| Dialysis therapy | 546 (2.82%) | 711 (4.99%) | 144 (8.31%) |
| Clinical outcome |  |  |  |
| Hospital mortality | 3082 (15.92%) | 2056 (14.44%) | 327 (18.88%) |
| Survival time (days) | 9.94 (6.84-15.47) | 10.80 (7.06-17.63) | 21.00 (12.00-28.00) |

ICU: intensive care unit; MICU: medical ICU; SICU: surgical ICU; ARDS: acute respiratory distress syndrome; SOFA: sequential organ failure assessment; APS-III: acute physiology score III; WBC: white blood cell.

^1^ Including sickle cell disease, thalassemia, vitamin B12 deficiency anemia, megaloblastic anemia, immunologic thrombocytopenic purpura, aplastic anemia

^2^ Including disseminated intravascular coagulation, Behcet’s disease, systemic lupus erythematosus, antiphospholipid syndrome, inflammatory bowel diseases

^3^ Including ischemic heart disease, ischemic stroke, deep-vein thrombosis, and pulmonary embolism

^4^ Described in patients with platelet transfusion

# **Supplementary Table 2.** Description of laboratory test results in the first 24 hours of ICU hospitalization for eligible patients in the eICU-CRD, MIMIC-IV, and MEARDS databases

| **Variable** | **Level** | **eICU-CRD**  **(*n* = 19,361)** | **MIMIC-IV**  **(*n* = 14,239)** | **MEARDS**  **(*n* = 1,732)** |
| --- | --- | --- | --- | --- |
| Platelets (10^9^/L) | Lowest | 173.00 (119.00-233.00) | 168.00 (115.00-231.00) | 184.00 (117.00-270.00) |
|  | Highest | 204.00 (149.00-269.00) | 208.00 (153.00-277.00) | - |
| INR (%) | Lowest | 1.20 (1.10-1.44) | 1.20 (1.10-1.40) | - |
|  | Highest | 1.30 (1.10-1.60) | 1.30 (1.15-1.60) | - |
| PTT (s) | Lowest | 30.60 (26.80-36.00) | 28.80 (25.80-33.50) | - |
|  | Highest | 33.00 (28.00-43.00) | 33.20 (28.30-46.70) | - |
| BUN (mg/dL) | Lowest | 19.00 (13.00-32.33) | 18.00 (12.00-29.00) | - |
|  | Highest | 23.00 (15.00-38.00) | 22.00 (15.00-35.00) | 26.00 (16.00-43.00) |
| Creatinine (mg/dL) | Lowest | 1.00 (0.73-1.57) | 0.90 (0.70-1.40) | 1.10 (0.79-1.80) |
|  | Highest | 1.20 (0.84-1.90) | 1.10 (0.80-1.70) | 1.34 (0.90-2.30) |
| Bilirubin (mg/dL) | Lowest | 0.60 (0.40-1.10) | 0.70 (0.40-1.30) | - |
|  | Highest | 0.70 (0.50-1.30) | 0.80 (0.40-1.50) | 0.80 (0.50-1.80) |
| ALT (IU/L) | Lowest | 27.00 (17.00-51.00) | 28.00 (16.00-61.00) | - |
|  | Highest | 30.00 (18.00-60.00) | 30.00 (18.00-74.00) | - |
| AST (IU/L) | Lowest | 35.00 (21.00-74.00) | 41.00 (24.00-90.00) | - |
|  | Highest | 40.00 (23.00-93.00) | 47.00 (26.00-123.00) | - |
| ALP (IU/L) | Lowest | 75.00 (56.00-107.00) | 76.00 (56.00-109.00) | - |
|  | Highest | 82.00 (60.00-117.00) | 82.00 (61.00-121.00) | - |
| WBC (10^9^/L) | Lowest | 10.50 (7.50-14.30) | 9.80 (7.00-13.20) | 11.70 (7.70-17.00) |
|  | Highest | 13.40 (9.60-18.50) | 13.60 (9.90-18.60) | 16.30 (11.50-22.70) |
| Bands (10^9^/L) | Lowest | 7.00 (3.00-15.00) | 2.00 (0.00-7.00) | - |
|  | Highest | 10.00 (4.00-19.00) | 3.00 (1.00-10.00) | - |
| Sodium (mmol/L) | Lowest | 137.00 (134.00-140.00) | 137.00 (134.00-140.00) | 136.00 (133.00-139.00) |
|  | Highest | 140.00 (137.00-143.00) | 140.00 (137.00-143.00) | 139.00 (136.00-142.00) |
| Potassium (mmol/L) | Lowest | 3.80 (3.40-4.20) | 3.90 (3.50-4.20) | 3.70 (3.30-4.10) |
|  | Highest | 4.30 (3.90-4.90) | 4.50 (4.10-5.00) | 4.40 (4.00-4.80) |
| Calcium (mg/dL) | Lowest | 8.00 (7.40-8.50) | 8.10 (7.50-8.60) | - |
|  | Highest | 8.50 (8.00-9.00) | 8.50 (8.10-9.00) | - |
| Chloride (mmol/L) | Lowest | 103.00 (99.00-107.00) | 103.00 (99.00-106.00) | - |
|  | Highest | 106.00 (102.00-111.00) | 106.00 (102.00-110.00) | - |
| Hematocrit (%) | Lowest | 31.40 (26.00-36.70) | 29.90 (25.20-35.20) | 29.50 (26.40-33.50) |
|  | Highest | 35.40 (30.50-40.50) | 34.90 (30.70-39.70) | 33.30 (30.03-37.40) |
| Hemoglobin (g/dL) | Lowest | 10.30 (8.60-12.20) | 9.90 (8.40-11.70) | - |
|  | Highest | 11.70 (10.00-13.50) | 11.50 (10.00-13.10) | - |
| Lactate (IU/L) | Lowest | 1.50 (1.03-2.30) | 1.40 (1.00-1.90) | - |
|  | Highest | 2.30 (1.40-4.20) | 2.30 (1.50-3.80) | - |
| Albumin (g/L) | Lowest | 2.80 (2.30-3.30) | 3.10 (2.60-3.60) | 2.40 (2.00-2.90) |
|  | Highest | 3.00 (2.50-3.50) | 3.30 (2.70-3.70) | 2.60 (2.10-3.00) |
| Glucose (mg/dL) | Lowest | 106.00 (88.00-127.00) | 114.00 (96.00-137.00) | 116.00 (96.00-141.75) |
|  | Highest | 170.00 (134.00-224.00) | 148.00 (120.00-194.00) | 177.00 (141.00-237.00) |
| Bicarbonate (mmol/L) | Lowest | 22.00 (19.00-25.00) | 21.00 (19.00-24.00) | 20.70 (17.00-24.30) |
|  | Highest | 24.00 (22.00-27.00) | 24.00 (22.00-27.00) | - |
| Total CO_2_ (mmHg) | Lowest | 22.20 (19.00-26.40) | 22.00 (19.00-25.00) | - |
|  | Highest | 26.00 (22.00-30.00) | 26.00 (24.00-29.00) | - |

BUN: blood urea nitrogen; INR: international normalized ratio; PTT: partial thromboplastin time; WBC: white blood cell; ALT: alanine aminotransferase; AST: aspartate aminotransferase; ALP: alkaline phosphatase.

# **Supplementary Table 3.** Description of vital signs in the first 24 hours of ICU hospitalization for eligible patients in the eICU-CRD, MIMIC-IV, and MEARDS databases

| **Variable** | **Level** | **eICU-CRD**  **(*n* = 19361)** | **MIMIC-IV**  **(*n* = 14239)** | **MEARDS**  **(*n* = 1732)** |
| --- | --- | --- | --- | --- |
| Heart rate (/min) | Lowest | 72 (61-85) | 70 (60-81) | 79 (65-89) |
|  | Highest | 110 (94-126) | 104 (90-120) | 115 (100-132) |
| Respiratory rate (/min) | Lowest | 13 (10-16) | 12 (10-15) | 14 (12-18) |
|  | Highest | 28 (24-34) | 27 (24-32) | 28 (24-34) |
| SpO_2_ (%) | Lowest | 92.00 (88.00-95.00) | 93.00 (90.00-95.00) | - |
|  | Highest | 100.00 (100.00-100.00) | 100.00 (100.00-100.00) | - |
| Temperature (ºC) | Lowest | 36.30 (35.90-36.60) | 36.44 (36.06-36.72) | 36.44 (36.00-36.89) |
|  | Highest | 37.40 (37.00-38.00) | 37.39 (37.00-37.94) | 38.17 (37.56-38.83) |
| DBP (mmHg) | Lowest | 46.00 (39.00-54.00) | 45.00 (39.00-52.00) | - |
|  | Highest | 85.00 (72.00-99.00) | 85.00 (74.00-98.00) | - |
| SBP (mmHg) | Lowest | 90.00 (79.00-104.00) | 88.00 (79.00-99.00) | 81.00 (73.00-90.00) |
|  | Highest | 150.00 (133.00-169.00) | 147.00 (133.00-163.00) | 141.00 (128.00-156.00) |
| MBP (mmHg) | Lowest | 61.00 (52.00-70.00) | 58.00 (51.00-65.00) | 58.00 (52.00-62.00) |
|  | Highest | 102.00 (90.00-117.00) | 102.00 (91.00-116.00) | 96.00 (87.00-107.00) |

SpO_2_: pulse oxygen saturation; DBP: diastolic blood pressure; SBP: systolic blood pressure; MBP: mean blood pressure.

# **Supplementary Table 4.** Description of Charlson comorbidities for eligible patients in the eICU-CRD and MIMIC-IV databases

| **Charlson comorbidity** | **eICU-CRD**  **(*n* = 19361)** | **MIMIC-IV**  **(*n* = 14239)** |
| --- | --- | --- |
| Myocardial infarct | 2445 (12.63%) | 2640 (18.54%) |
| Congestive heart failure | 3992 (20.62%) | 4490 (31.53%) |
| Peripheral vascular disease | 1154 (5.96%) | 1803 (12.66%) |
| Cerebrovascular disease | 3194 (16.50%) | 2926 (20.55%) |
| Dementia | 758 (3.92%) | 486 (3.41%) |
| Chronic pulmonary disease | 3588 (18.53%) | 3838 (26.95%) |
| Rheumatic disease | 460 (2.38%) | 517 (3.63%) |
| Peptic ulcer disease | 577 (2.98%) | 424 (2.98%) |
| Mild liver disease | 1046 (5.40%) | 1936 (13.60%) |
| Diabetes | 5427 (28.03%) | 4159 (29.21%) |
| Paraplegia | 90 (0.46%) | 1085 (7.62%) |
| Renal disease | 3107 (16.05%) | 3007 (21.12%) |
| Malignant cancer | 2862 (14.78%) | 1812 (12.73%) |
| Severe liver disease | 580 (3.00%) | 911 (6.40%) |
| Metastatic solid tumor | 522 (2.70%) | 837 (5.88%) |
| AIDS | 36 (0.19%) | 84 (0.59%) |

AIDS: acquired immune deficiency syndrome.

# **Supplementary Table 5.** Description of chronic comorbidities for eligible patients in the MEARDS database

| **Chronic comorbidity** | **MEARDS**  **(*n* = 1732)** |
| --- | --- |
| Non-Hodgkin’s lymphoma | 8 (0.46%) |
| AIDS | 0 |
| Leukemia | 0 |
| Solid tumor with metastasis | 40 (2.31%) |
| Immune suppression | 137 (7.91%) |
| Hepatic failure with coma | 61 (3.52%) |
| Cirrhosis | 88 (5.08%) |
| Diabetes | 447 (25.81%) |

AIDS: acquired immune deficiency syndrome.

# **Supplementary Table 6.** Summary statistics describing the features of individual dynamic platelet trajectories

| **Index** | **Summary statistics** |
| --- | --- |
| 1 | Range |
| 2 | Mean-over-time |
| 3 | Standard deviation (SD) |
| 4 | Coefficient of variation (CV) |
| 5 | Change |
| 6 | Mean change per unit time |
| 7 | Change relative to the first score |
| 8 | Change relative to the mean over time |
| 9 | Slope of the linear model |
| 10 | *R*^2^: Proportion of variance explained by the linear model |
| 11 | Maximum of the first differences |
| 12 | SD of the first differences |
| 13 | SD of the first differences per time unit |
| 14 | Mean of the absolute first differences |
| 15 | Maximum of the absolute first differences |
| 16 | Ratio of the maximum absolute difference to the mean-over-time |
| 17 | Ratio of the maximum absolute first difference to the slope |
| 18 | Ratio of the SD of the first differences to the slope |
| 19 | Mean of the second differences |
| 20 | Mean of the absolute second differences |
| 21 | Maximum of the absolute second differences |
| 22 | Ratio of the maximum absolute second difference to the mean-over-time |
| 23 | Ratio of the maximum absolute second difference to mean absolute first difference |
| 24 | Ratio of the mean absolute second difference to the mean absolute first difference |

# **Supplementary Table 7.** Statistics of four models with different numbers of dynamic platelet count trajectory patterns in the discovery set

| **Number of trajectory patterns** | **-****2*Log-likelihood** | **AIC** | **BIC** |
| --- | --- | --- | --- |
| 1 | 53283.10 | 53283.10 | 53283.10 |
| 2 | 53147.34 | 53149.34 | 53155.31 |
| 3 | 53121.48 | 53125.48 | 53137.43 |
| 4 | 53127.14 | 53133.14 | 53151.07 |

AIC: Akaike information criterion; BIC: Bayesian information criteria.

The best number of trajectory patterns is 3, which yields the lowest -2*log-likelihood, AIC and BIC for the statistical model.

# **Supplementary Table 8.** Comparison of demographics, severity scores, and treatment supports across the three dynamic platelet count trajectory patterns in the eICU-CRD database

| **Variable** | **Ascending**  **(*n* = 4361)** | **Stable**  **(*n* = 12106)** | **Descending**  **(*n* = 2894)** | ***P*** |
| --- | --- | --- | --- | --- |
| Age (years) | 64.00 (52.00-76.00) | 67.00 (55.00-77.00) | 66.00 (55.00-76.00) | < 0.001 |
| Gender, male | 2434 (55.81%) | 6902 (57.01%) | 1488 (51.42%) | < 0.001 |
| Ethnicity |  |  |  | 0.2500 |
| Caucasian | 3306 (75.81%) | 9226 (76.21%) | 2221 (76.74%) |  |
| African American | 490 (11.24%) | 1372 (11.33%) | 302 (10.44%) |  |
| Asian | 89 (2.04%) | 209 (1.73%) | 50 (1.73%) |  |
| Hispanic | 147 (3.37%) | 495 (4.09%) | 111 (3.84%) |  |
| Native American | 27 (0.62%) | 65 (0.54%) | 15 (0.52%) |  |
| Other/Unknown | 302 (6.93%) | 739 (6.10%) | 195 (6.74%) |  |
| First ICU location |  |  |  | < 0.001 |
| Cardiac ICU | 989 (22.68%) | 2602 (21.49%) | 695 (24.02%) |  |
| Med-Surg ICU | 2268 (52.01%) | 6351 (52.46%) | 1553 (53.66%) |  |
| MICU | 358 (8.21%) | 1061 (8.76%) | 286 (9.88%) |  |
| Neuro ICU | 315 (7.22%) | 1098 (9.07%) | 179 (6.19%) |  |
| SICU | 431 (9.88%) | 994 (8.21%) | 181 (6.25%) |  |
| ARDS at baseline |  |  |  | < 0.001 |
| Mild | 220 (5.04%) | 656 (5.42%) | 154 (5.32%) |  |
| Moderate | 338 (7.75%) | 879 (7.26%) | 230 (7.95%) |  |
| Severe | 142 (3.26%) | 516 (4.26%) | 189 (6.53%) |  |
| Sepsis at baseline | 879 (20.16%) | 2463 (20.35%) | 885 (30.58%) | < 0.001 |
| Immunotherapy | 16 (0.37%) | 65 (0.54%) | 12 (0.41%) | 0.3253 |
| Hematologic diseases^1^ | 235 (5.39%) | 591 (4.88%) | 127 (4.39%) | 0.1472 |
| Thromboinflammatory diseases^2^ | 151 (3.46%) | 483 (3.99%) | 133 (4.60%) | 0.0513 |
| Thrombotic diseases^3^ | 665 (15.25%) | 2233 (18.45%) | 615 (21.25%) | < 0.001 |
| Severity of illness | | | | |
| SOFA | 6.00 (4.00-8.00) | 6.00 (4.00-9.00) | 6.00 (4.00-9.00) | < 0.001 |
| APS-III | 46.00 (34.00-63.00) | 50.00 (36.00-70.00) | 61.00 (42.00-82.00) | < 0.001 |
| Platelet treatment |  |  |  |  |
| Platelet transfusion | 224 (5.14%) | 719 (5.94%) | 117 (4.04%) | < 0.001 |
| Platelet amount^4^ | 448.00 (258.00 - 708.00) | 450.00 (255.50 - 701.00) | 314.00 (249.94 - 584.25) | 0.1550 |
| Antiplatelet treatment | 601 (13.78%) | 1718 (14.19%) | 431 (14.89%) | 0.4131 |
| Support within the first 24 h | | | | |
| Mechanical ventilation | 2192 (50.26%) | 6065 (50.10%) | 1519 (52.49%) | 0.0655 |
| Vasopressor | 670 (15.36%) | 2521 (20.82%) | 905 (31.27%) | < 0.001 |
| Dialysis therapy | 110 (2.52%) | 353 (2.92%) | 83 (2.87%) | 0.4027 |

ICU: intensive care unit; MICU: medical ICU; SICU: surgical ICU; ARDS: acute respiratory distress syndrome; SOFA: sequential organ failure assessment; APS-III: acute physiology score III; WBC: white blood cell.

^1^ Including sickle cell disease, thalassemia, vitamin B12 deficiency anemia, megaloblastic anemia, immunologic thrombocytopenic purpura, aplastic anemia

^2^ Including disseminated intravascular coagulation, Behcet’s disease, systemic lupus erythematosus, antiphospholipid syndrome, inflammatory bowel diseases

^3^ Including ischemic heart disease, ischemic stroke, deep-vein thrombosis, and pulmonary embolism

^4^ Described in patients with platelet transfusion

# **Supplementary Table 9.** Comparison of laboratory tests in the first 24 hours of ICU hospitalization across the three dynamic platelet count trajectory patterns in the eICU-CRD database

| **Variable** | | **Ascending**  **(*n* = 4361)** | **Stable**  **(*n* = 12106)** | **Descending**  **(*n* = 2894)** | ***P*** |
| --- | --- | --- | --- | --- | --- |
| Platelets (10^9^/L) | Lowest | 162.00 (109.00-220.00) | 162.00 (111.00-216.00) | 244.00 (191.00-314.00) | < 0.001 |
|  | Highest | 196.00 (143.00-259.00) | 191.00 (139.00-250.00) | 278.00 (220.00-363.00) | < 0.001 |
| BUN (mg/dL) | Lowest | 17.00 (11.00-27.00) | 20.00 (13.00-33.00) | 23.00 (14.00-39.00) | < 0.001 |
|  | Highest | 20.00 (13.00-32.00) | 23.00 (15.00-39.00) | 27.00 (17.00-46.00) | < 0.001 |
| Creatinine (mg/dL) | Lowest | 0.90 (0.68-1.31) | 1.01 (0.74-1.59) | 1.12 (0.79-1.91) | < 0.001 |
|  | Highest | 1.05 (0.76-1.60) | 1.20 (0.86-1.91) | 1.40 (0.91-2.48) | < 0.001 |
| WBC (10^9^/L) | Lowest | 10.30 (7.50-13.80) | 10.10 (7.20-13.70) | 12.80 (9.19-18.30) | < 0.001 |
|  | Highest | 13.12 (9.60-17.80) | 12.90 (9.20-17.80) | 16.50 (11.60-23.00) | < 0.001 |
| Sodium (mmol/L) | Lowest | 137.00 (134.00-140.00) | 137.00 (134.00-140.00) | 137.00 (133.00-140.00) | < 0.001 |
|  | Highest | 139.00 (136.00-142.00) | 140.00 (137.00-143.00) | 140.00 (137.00-143.00) | < 0.001 |
| Potassium (mmol/L) | Lowest | 3.80 (3.40-4.10) | 3.80 (3.40-4.20) | 3.80 (3.40-4.20) | < 0.001 |
|  | Highest | 4.30 (3.90-4.70) | 4.30 (3.90-4.90) | 4.40 (4.00-5.00) | < 0.001 |
| Calcium (mg/dL) | Lowest | 7.90 (7.40-8.40) | 8.00 (7.50-8.60) | 7.90 (7.20-8.50) | < 0.001 |
|  | Highest | 8.40 (7.90-8.90) | 8.50 (8.00-9.10) | 8.50 (7.90-9.10) | < 0.001 |
| Chloride (mmol/L) | Lowest | 103.00 (99.00-107.00) | 103.00 (99.00-107.00) | 103.00 (98.00-107.00) | 0.0010 |
|  | Highest | 106.00 (102.00-110.00) | 107.00 (102.00-111.00) | 107.00 (102.00-111.00) | < 0.001 |
| Hematocrit (%) | Lowest | 29.50 (25.00-34.30) | 32.00 (26.20-37.10) | 32.40 (27.10-38.30) | < 0.001 |
|  | Highest | 33.90 (29.50-38.40) | 35.80 (31.00-41.00) | 36.20 (31.00-41.70) | < 0.001 |
| Hemoglobin (g/dL) | Lowest | 9.80 (8.20-11.40) | 10.50 (8.60-12.30) | 10.60 (8.80-12.70) | < 0.001 |
|  | Highest | 11.20 (9.80-12.90) | 11.90 (10.10-13.60) | 11.80 (10.10-13.90) | < 0.001 |
| Glucose (mg/dL) | Lowest | 105.00 (89.00-125.00) | 106.00 (88.00-127.00) | 107.00 (87.00-131.00) | 0.1987 |
|  | Highest | 164.00 (132.00-211.00) | 170.00 (135.00-223.00) | 181.00 (138.00-253.50) | < 0.001 |
| Bicarbonate (mmol/L) | Lowest | 23.00 (20.00-25.00) | 22.00 (19.00-25.00) | 21.00 (17.00-24.00) | < 0.001 |
|  | Highest | 25.00 (22.00-27.00) | 25.00 (22.00-27.00) | 23.00 (20.50-26.00) | < 0.001 |

BUN: blood urea nitrogen; WBC: white blood cell.

# **Supplementary Table 10.** Comparison of vital signs in the first 24 hours of ICU hospitalization across the three dynamic platelet count trajectory patterns in the eICU-CRD database

| **Variable** | | **Ascending**  **(*n* = 4361)** | **Stable**  **(*n* = 12106)** | **Descending**  **(*n* = 2894)** | ***P*** |
| --- | --- | --- | --- | --- | --- |
| Heart rate (/min) | Lowest | 74 (63-85) | 71 (60-83) | 75 (62-88) | < 0.001 |
|  | Highest | 110 (96-127) | 108 (93-125) | 115 (99-132) | < 0.001 |
| Respiratory rate (/min) | Lowest | 13 (11-16) | 13 (10-16) | 13 (10-16) | < 0.001 |
|  | Highest | 28 (24-34) | 28 (23-34) | 29 (24-35) | < 0.001 |
| SpO_2_ (%) | Lowest | 92.00 (88.00-95.00) | 92.00 (88.00-95.00) | 91.00 (86.00-95.00) | < 0.001 |
|  | Highest | 100.00 (99.00-100.00) | 100.00 (100.00-100.00) | 100.00 (100.00-100.00) | < 0.001 |
| Temperature (ºC) | Lowest | 36.40 (36.00-36.70) | 36.30 (35.90-36.60) | 36.30 (35.80-36.60) | < 0.001 |
|  | Highest | 37.56 (37.10-38.20) | 37.40 (37.00-38.00) | 37.40 (36.90-38.00) | < 0.001 |
| DBP (mmHg) | Lowest | 48.00 (40.00-55.00) | 46.00 (39.00-54.00) | 45.00 (37.00-53.00) | < 0.001 |
|  | Highest | 85.00 (73.00-98.00) | 85.00 (72.00-100.00) | 85.00 (71.00-98.00) | 0.0213 |
| SBP (mmHg) | Lowest | 92.00 (81.00-106.00) | 91.00 (79.00-104.00) | 86.00 (74.00-99.00) | < 0.001 |
|  | Highest | 150.00 (134.00-169.00) | 151.00 (134.00-170.00) | 145.00 (129.00-165.00) | < 0.001 |
| MBP (mmHg) | Lowest | 62.00 (54.00-71.00) | 61.00 (52.00-70.00) | 59.00 (50.00-68.00) | < 0.001 |
|  | Highest | 102.00 (90.50-117.00) | 103.00 (90.00-118.00) | 101.00 (88.00-115.50) | < 0.001 |

SpO_2_: pulse oxygen saturation; DBP: diastolic blood pressure; SBP: systolic blood pressure; MBP: mean blood pressure.

# **Supplementary Table 11.** Comparison of Charlson comorbidities across the three dynamic platelet count trajectory patterns in the eICU-CRD database

| **Comorbidity** | **Ascending**  **(*n* = 4361)** | **Stable**  **(*n* = 12106)** | **Descending**  **(*n* = 2894)** | ***P*** |
| --- | --- | --- | --- | --- |
| Myocardial infarct | 460 (10.55%) | 1527 (12.61%) | 458 (15.83%) | < 0.001 |
| Congestive heart failure | 776 (17.79%) | 2628 (21.71%) | 588 (20.32%) | < 0.001 |
| Peripheral vascular disease | 246 (5.64%) | 725 (5.99%) | 183 (6.32%) | 0.4743 |
| Cerebrovascular disease | 619 (14.19%) | 2148 (17.74%) | 427 (14.75%) | <0.001 |
| Dementia | 162 (3.71%) | 470 (3.88%) | 126 (4.35%) | 0.3715 |
| Chronic pulmonary disease | 727 (16.67%) | 2317 (19.14%) | 544 (18.80%) | 0.0014 |
| Rheumatic disease | 114 (2.61%) | 267 (2.21%) | 79 (2.73%) | 0.1259 |
| Peptic ulcer disease | 123 (2.82%) | 358 (2.96%) | 96 (3.32%) | 0.4621 |
| Mild liver disease | 149 (3.42%) | 726 (6.00%) | 171 (5.91%) | < 0.001 |
| Diabetes | 1100 (25.22%) | 3436 (28.38%) | 891 (30.79%) | < 0.001 |
| Paraplegia | 23 (0.53%) | 50 (0.41%) | 17 (0.59%) | 0.3658 |
| Renal disease | 595 (13.64%) | 2032 (16.79%) | 480 (16.59%) | < 0.001 |
| Malignant cancer | 568 (13.02%) | 1867 (15.42%) | 427 (14.75%) | < 0.001 |
| Severe liver disease | 70 (1.61%) | 428 (3.54%) | 82 (2.83%) | < 0.001 |
| Metastatic solid tumor | 99 (2.27%) | 310 (2.56%) | 113 (3.90%) | < 0.001 |
| AIDS | 7 (0.16%) | 21 (0.17%) | 8 (0.28%) | 0.4401 |

AIDS: acquired immune deficiency syndrome.

# **Supplementary Table 12.** Comparison of demographics, severity scores, and treatment supports across three dynamic platelet count trajectory patterns in the MIMIC-IV database

| **Variable** | **Ascending**  **(*n* = 2872)** | **Stable**  **(*n* = 10113)** | **Descending**  **(*n* = 1254)** | ***P*** |
| --- | --- | --- | --- | --- |
| Age (years) | 65.72 (53.32-76.72) | 68.21 (56.27-78.91) | 67.32 (54.98-77.50) | < 0.001 |
| Gender, male | 1587 (55.26%) | 5913 (58.47%) | 611 (48.72%) | < 0.001 |
| Ethnicity |  |  |  | 0.0075 |
| African American | 205 (7.14%) | 885 (8.75%) | 87 (6.94%) |  |
| Asian | 78 (2.72%) | 275 (2.72%) | 25 (1.99%) |  |
| Caucasian | 1913 (66.61%) | 6582 (65.08%) | 841 (67.07%) |  |
| Hispanic | 110 (3.83%) | 307 (3.04%) | 34 (2.71%) |  |
| Native American | 9 (0.31%) | 22 (0.22%) | 0 (0.00%) |  |
| Other/Unknown | 557 (19.39%) | 2042 (20.19%) | 267 (21.29%) |  |
| First ICU location |  |  |  | < 0.001 |
| Cardiac ICU | 967 (33.67%) | 2951 (29.18%) | 349 (27.83%) |  |
| Med-Surg ICU | 424 (14.76%) | 1351 (13.36%) | 214 (17.07%) |  |
| MICU | 479 (16.68%) | 1807 (17.87%) | 275 (21.93%) |  |
| Neuro ICU | 125 (4.35%) | 934 (9.24%) | 50 (3.99%) |  |
| SICU | 877 (30.54%) | 3070 (30.36%) | 366 (29.19%) |  |
| ARDS at baseline |  |  |  | < 0.001 |
| Mild | 144 (5.01%) | 502 (4.96%) | 49 (3.91%) |  |
| Moderate | 225 (7.83%) | 721 (7.13%) | 112 (8.93%) |  |
| Severe | 93 (3.24%) | 401 (3.97%) | 106 (8.45%) |  |
| Sepsis at baseline | 1742 (60.65%) | 6060 (59.92%) | 905 (72.17%) |  |
| Immunotherapy | 25 (0.87%) | 140 (1.38%) | 10 (0.80%) | 0.0306 |
| Hematologic diseases^1^ | 64 (2.23%) | 211 (2.09%) | 31 (2.47%) | 0.6386 |
| Thromboinflammatory diseases^2^ | 154 (5.36%) | 575 (5.69%) | 108 (8.61%) | < 0.001 |
| Thrombotic diseases^3^ | 869 (30.26%) | 3197 (31.61%) | 457 (36.44%) | < 0.001 |
| Severity of illness | | | | |
| SOFA | 6.00 (3.00-8.00) | 6.00 (4.00-9.00) | 7.00 (4.00-11.00) | < 0.001 |
| APS-III | 47.00 (35.00-66.00) | 52.00 (37.00-73.00) | 64.00 (46.00-87.00) | < 0.001 |
| Platelet treatment |  |  |  |  |
| Platelet transfusion | 346 (12.05%) | 1381 (13.66%) | 121 (9.65%) | < 0.001 |
| Platelet amount^4^ | 423.00 (243.00 - 693.00) | 454.00 (267.75 - 891.50) | 334.00 (228.00 - 571.00) | < 0.001 |
| Antiplatelet treatment | 588 (20.47%) | 2063 (20.40%) | 233 (18.58%) | 0.3023 |
| Support within the first 24 h | | | | |
| Mechanical ventilation | 1594 (55.50%) | 5466 (54.05%) | 694 (55.34%) | 0.3107 |
| Vasopressor | 110 (3.83%) | 756 (7.48%) | 179 (14.27%) | < 0.001 |
| Dialysis therapy | 92 (3.20%) | 519 (5.13%) | 100 (7.97%) | < 0.001 |

ICU: intensive care unit; MICU: medical ICU; SICU: surgical ICU; ARDS: acute respiratory distress syndrome; SOFA: sequential organ failure assessment; APS-III: acute physiology score III; WBC: white blood cell.

^1^ Including sickle cell disease, thalassemia, vitamin B12 deficiency anemia, megaloblastic anemia, immunologic thrombocytopenic purpura, aplastic anemia

^2^ Including disseminated intravascular coagulation, Behcet’s disease, systemic lupus erythematosus, antiphospholipid syndrome, inflammatory bowel diseases

^3^ Including ischemic heart disease, ischemic stroke, deep-vein thrombosis, and pulmonary embolism

^4^ Described in patients with platelet transfusion

# **Supplementary Table 13.** Comparison of laboratory tests in the first 24 hours of ICU hospitalization across the three dynamic platelet count trajectory patterns in the MIMIC-IV database

| **Variable** | | **Ascending**  **(*n* = 2872)** | **Stable**  **(*n* = 10113)** | **Descending**  **(*n* = 1254)** | ***P*** |
| --- | --- | --- | --- | --- | --- |
| Platelets (10^9^/L) | Lowest | 163.00 (112.00-229.00) | 161.00 (110.00-218.00) | 260.00 (197.00-343.00) | < 0.001 |
|  | Highest | 208.00 (154.00-283.00) | 199.00 (146.00-259.00) | 313.00 (246.00-413.00) | < 0.001 |
| INR (%) | Lowest | 1.20 (1.10-1.30) | 1.20 (1.10-1.40) | 1.20 (1.10-1.50) | < 0.001 |
|  | Highest | 1.30 (1.20-1.60) | 1.30 (1.10-1.70) | 1.30 (1.10-1.70) | 0.0548 |
| PTT (s) | Lowest | 28.60 (25.80-32.40) | 28.80 (25.70-33.70) | 29.50 (25.92-35.58) | < 0.001 |
|  | Highest | 32.60 (28.40-41.30) | 33.30 (28.30-47.30) | 35.25 (28.70-58.80) | < 0.001 |
| BUN (mg/dL) | Lowest | 15.00 (11.00-24.00) | 18.00 (13.00-30.00) | 21.00 (13.00-35.00) | < 0.001 |
|  | Highest | 19.00 (13.00-29.00) | 22.00 (15.00-36.00) | 25.00 (17.00-42.00) | < 0.001 |
| Creatinine (mg/dL) | Lowest | 0.80 (0.60-1.20) | 0.90 (0.70-1.40) | 1.00 (0.70-1.60) | < 0.001 |
|  | Highest | 1.00 (0.70-1.40) | 1.10 (0.80-1.80) | 1.20 (0.80-2.10) | < 0.001 |
| WBC (10^9^/L) | Lowest | 9.70 (7.00-13.00) | 9.50 (6.90-12.90) | 12.00 (8.95-16.65) | < 0.001 |
|  | Highest | 13.60 (10.00-18.50) | 13.30 (9.60-18.00) | 16.90 (12.20-23.75) | < 0.001 |
| Sodium (mmol/L) | Lowest | 137.00 (134.00-139.00) | 137.00 (135.00-140.00) | 137.00 (133.00-139.00) | < 0.001 |
|  | Highest | 140.00 (137.00-142.00) | 140.00 (138.00-143.00) | 140.00 (137.00-142.00) | < 0.001 |
| Potassium (mmol/L) | Lowest | 3.90 (3.50-4.20) | 3.90 (3.50-4.30) | 3.90 (3.50-4.40) | < 0.001 |
|  | Highest | 4.40 (4.00-4.80) | 4.50 (4.10-5.00) | 4.60 (4.20-5.20) | < 0.001 |
| Calcium (mg/dL) | Lowest | 8.00 (7.50-8.40) | 8.10 (7.60-8.70) | 7.90 (7.30-8.50) | < 0.001 |
|  | Highest | 8.40 (7.90-8.80) | 8.60 (8.10-9.10) | 8.50 (8.10-9.00) | < 0.001 |
| Chloride (mmol/L) | Lowest | 103.00 (99.00-106.00) | 103.00 (99.00-106.00) | 102.00 (98.00-105.00) | < 0.001 |
|  | Highest | 107.00 (103.00-110.00) | 106.00 (102.00-110.00) | 106.00 (102.00-110.00) | 0.0019 |
| Hematocrit (%) | Lowest | 26.00 (22.00-31.00) | 28.00 (23.00-34.00) | 30.00 (25.00-35.00) | < 0.001 |
|  | Highest | 35.00 (31.00-39.00) | 35.00 (31.00-39.00) | 34.00 (29.00-38.00) | 0.0033 |
| Hemoglobin (g/dL) | Lowest | 8.80 (7.40-10.20) | 9.30 (7.70-11.30) | 10.10 (8.30-11.80) | < 0.001 |
|  | Highest | 11.60 (10.30-13.00) | 11.70 (10.30-13.10) | 11.30 (9.70-12.70) | 0.0036 |
| Glucose (mg/dL) | Lowest | 112.00 (95.00-132.00) | 114.00 (96.00-137.00) | 116.50 (94.00-143.00) | < 0.001 |
|  | Highest | 139.00 (115.00-178.00) | 149.00 (121.00-195.00) | 161.00 (130.00-216.00) | < 0.001 |
| Bicarbonate (mmol/L) | Lowest | 22.00 (20.00-24.00) | 21.00 (19.00-24.00) | 20.00 (16.00-23.00) | < 0.001 |
|  | Highest | 24.00 (22.00-27.00) | 24.00 (22.00-26.00) | 23.00 (20.00-26.00) | < 0.001 |

BUN: blood urea nitrogen; INR: international normalized ratio; PTT: partial thromboplastin time; WBC: white blood cell.

# **Supplementary Table 14.** Comparison of vital signs in the first 24 hours of ICU hospitalization across the three dynamic platelet count trajectory patterns in the MIMIC-IV database

| **Variable** | | **Ascending**  **(*n* = 2872)** | **Stable**  **(*n* = 10113)** | **Descending**  **(*n* = 1254)** | ***P*** |
| --- | --- | --- | --- | --- | --- |
| Heart rate (/min) | Lowest | 72 (62-83) | 69 (60-80) | 74 (62-87) | < 0.001 |
|  | Highest | 106 (93-122) | 102 (90-118) | 112 (95-125) | < 0.001 |
| Respiratory rate (/min) | Lowest | 13 (10-15) | 12 (10-15) | 13 (11-15) | < 0.001 |
|  | Highest | 28 (24-32) | 27 (24-32) | 29 (25-33) | < 0.001 |
| SpO_2_ (%) | Lowest | 93.00 (90.00-95.00) | 93.00 (90.00-95.00) | 92.00 (89.00-95.00) | < 0.001 |
|  | Highest | 100.00 (100.00-100.00) | 100.00 (100.00-100.00) | 100.00 (100.00-100.00) | 0.1759 |
| Temperature (ºC) | Lowest | 36.50 (36.06-36.78) | 36.44 (36.06-36.72) | 36.39 (35.89-36.67) | < 0.001 |
|  | Highest | 37.50 (37.11-38.20) | 37.33 (37.00-37.89) | 37.33 (36.94-37.95) | < 0.001 |
| DBP (mmHg) | Lowest | 45.00 (40.00-52.00) | 45.00 (39.00-52.00) | 44.00 (37.00-51.00) | < 0.001 |
|  | Highest | 84.00 (73.00-98.00) | 85.00 (74.00-98.00) | 86.00 (74.00-100.00) | 0.1114 |
| SBP (mmHg) | Lowest | 89.00 (80.00-98.00) | 89.00 (79.00-100.00) | 84.00 (76.00-94.00) | < 0.001 |
|  | Highest | 147.00 (134.00-163.00) | 147.00 (133.00-164.00) | 144.00 (129.00-161.00) | < 0.001 |
| MBP (mmHg) | Lowest | 58.00 (52.00-65.00) | 58.00 (51.00-66.00) | 56.00 (49.00-63.00) | < 0.001 |
|  | Highest | 102.00 (91.00-115.00) | 102.00 (92.00-116.00) | 102.50 (91.00-118.00) | 0.312 |

SpO_2_: pulse oxygen saturation; DBP: diastolic blood pressure; SBP: systolic blood pressure; MBP: mean blood pressure.

# **Supplementary Table 15.** Comparison of Charlson comorbidities across the three dynamic platelet count trajectory patterns in the MIMIC-IV database

| **Comorbidity** | **Ascending**  **(*n* = 2872)** | **Stable**  **(*n* = 10113)** | **Descending**  **(*n* = 1254)** | ***P*** |
| --- | --- | --- | --- | --- |
| Myocardial infarct | 501 (17.44%) | 1847 (18.26%) | 292 (23.29%) | < 0.001 |
| Congestive heart failure | 740 (25.77%) | 3310 (32.73%) | 440 (35.08%) | < 0.001 |
| Peripheral vascular disease | 341 (11.87%) | 1308 (12.93%) | 154 (12.28%) | 0.2928 |
| Cerebrovascular disease | 446 (15.53%) | 2267 (22.42%) | 213 (16.99%) | < 0.001 |
| Dementia | 86 (2.99%) | 358 (3.54%) | 42 (3.35%) | 0.3612 |
| Chronic pulmonary disease | 787 (27.40%) | 2708 (26.78%) | 343 (27.35%) | 0.7578 |
| Rheumatic disease | 116 (4.04%) | 342 (3.38%) | 59 (4.70%) | 0.0261 |
| Peptic ulcer disease | 85 (2.96%) | 303 (3.00%) | 36 (2.87%) | 0.9681 |
| Mild liver disease | 265 (9.23%) | 1486 (14.69%) | 185 (14.75%) | < 0.001 |
| Diabetes | 915 (31.86%) | 3260 (32.24%) | 406 (32.38%) | 0.9178 |
| Paraplegia | 148 (5.15%) | 860 (8.50%) | 77 (6.14%) | < 0.001 |
| Renal disease | 508 (17.69%) | 2239 (22.14%) | 260 (20.73%) | < 0.001 |
| Malignant cancer | 301 (10.48%) | 1332 (13.17%) | 179 (14.27%) | < 0.001 |
| Severe liver disease | 83 (2.89%) | 766 (7.57%) | 62 (4.94%) | < 0.001 |
| Metastatic solid tumor | 148 (5.15%) | 576 (5.70%) | 113 (9.01%) | < 0.001 |
| AIDS | 19 (0.66%) | 59 (0.58%) | 6 (0.48%) | 0.7694 |

AIDS: acquired immune deficiency syndrome.

# **Supplementary Table 16.** Associations between thrombocytopenia and 28-day mortality

| **Thrombocytopenia onset time** | **Model** | **eICU-CRD** | | | **MIMIC-IV** | | |
| --- | --- | --- | --- | --- | --- | --- | --- |
|  |  | ***HR*** | **95% CI** | ***P*** | ***HR*** | **95% CI** | ***P*** |
| At ICU admission | Univariate | 1.33 | (1.22-1.45) | 1.37×10^-10^ | 1.41 | (1.27-1.56) | 8.45×10^-11^ |
|  | Multivariable | 1.11 | (0.99-1.25) | 0.086 | 1.28 | (1.10-1.48) | 0.001 |
| Between 2-4 days^a^ | Univariate | 1.46 | (1.31-1.62) | 2.20×10^-12^ | 1.40 | (1.21-1.61) | 3.78×10^-6^ |
|  | Multivariable | 1.18 | (1.05-1.34) | 0.007 | 1.25 | (1.06-1.46) | 0.007 |
| Within 4 days | Univariate | 1.43 | (1.33-1.54) | 8.77×10^-21^ | 1.45 | (1.32-1.59) | 4.54×10^-15^ |
|  | Multivariable | 1.15 | (1.04-1.28) | 0.007 | 1.28 | (1.12-1.46) | 2.91×10^-4^ |
| Between 4-28 days^b^ | Univariate | 1.78 | (1.54-2.06) | 6.22×10^-15^ | 1.67 | (1.48-1.87) | 3.06×10^-17^ |
|  | Multivariable | 1.52 | (1.31-1.77) | 7.03×10^-8^ | 1.42 | (1.24-1.63) | 4.15×10^-7^ |
| Within 28 days | Univariate | 1.55 | (1.44-1.67) | 1.52×10^-31^ | 1.62 | (1.48-1.77) | 2.60×10^-25^ |
|  | Multivariable | 1.29 | (1.17-1.42) | 2.26×10^-7^ | 1.40 | (1.24-1.58) | 4.94×10^-8^ |

^a^ Excluded patients with thrombocytopenia onset at ICU admission

^b^ Excluded patients with thrombocytopenia onset within first 4 days of ICU hospitalization

Multivariable models were adjusted for age, gender, ethnicity, baseline platelet count, and antiplatelet treatment, platelet transfusion, transfusion amount, malignancies, hematologic diseases, immune therapy, thrombotic diseases, thromboinflammatory diseases, first ICU location, ARDS, sepsis, SOFA, APS-III, and supports within 24 hours (mechanical ventilation, vasopressor, and dialysis).

# **Supplementary Table 17.** Associations between platelet count trajectories and risk of thrombocytopenia

| **Model** | **Cluster** | **eICU-CRD** | | | **MIMIC-IV** | | | **Meta** | | |
| --- | --- | --- | --- | --- | --- | --- | --- | --- | --- | --- |
|  |  | ***OR*** | **95% CI** | ***P*** | ***OR*** | **95% CI** | ***P*** | ***OR*** | **95% CI** | ***P*** |
| Model_0_ | Ascending | Reference | | | Reference | | | Reference | | |
|  | Stable | 3.20 | (2.36-4.33) | 5.73×10^-14^ | 2.58 | (1.84-3.61) | 3.47×10^-8^ | 2.91 | (2.32-3.64) | 1.83×10^-20^ |
|  | Descending | 6.84 | (4.95-9.45) | 1.73×10^-31^ | 6.27 | (4.27-9.20) | 7.61×10^-21^ | 6.60 | (5.15-8.45) | 1.45×10^-50^ |
| Model_1_ | Ascending | Reference | | | Reference | | | Reference | | |
|  | Stable | 3.45 | (2.54-4.68) | 2.12×10^-15^ | 2.65 | (1.89-3.73) | 1.80×10^-8^ | 3.07 | (2.44-3.85) | 4.29×10^-22^ |
|  | Descending | 17.99 | (12.59-25.70) | 8.28×10^-57^ | 15.42 | (10.03-23.70) | 9.91×10^-36^ | 16.89 | (12.84-22.23) | 1.47×10^-90^ |
| Model_2_ | Ascending | Reference | | | Reference | | | Reference | | |
|  | Stable | 3.21 | (2.35-4.40) | 3.56×10^-13^ | 2.67 | (1.89-3.78) | 2.51×10^-8^ | 2.95 | (2.34-3.73) | 6.77×10^-20^ |
|  | Descending | 14.31 | (9.86-20.75) | 1.16×10^-44^ | 10.53 | (6.74-16.47) | 5.48×10^-25^ | 12.62 | (9.48-16.80) | 1.13×10^-67^ |
| Model_3_ | Ascending | Reference | | | Reference | | | Reference | | |
|  | Stable | 3.16 | (2.28-4.39) | 5.93×10^-12^ | 2.55 | (1.79-3.63) | 2.11×10^-7^ | 2.86 | (2.25-3.64) | 9.73×10^-18^ |
|  | Descending | 13.88 | (9.39-20.51) | 8.42×10^-40^ | 9.75 | (6.15-15.46) | 3.50×10^-22^ | 11.97 | (8.89-16.13) | 6.08×10^-60^ |
| Model_4_ | Ascending | Reference | | | Reference | | | Reference | | |
|  | Stable | 3.30 | (2.32-4.69) | 3.21×10^-11^ | 2.55 | (1.76-3.69) | 8.36×10^-7^ | 2.92 | (2.26-3.77) | 1.81×10^-16^ |
|  | Descending | 15.38 | (10.14-23.35) | 9.45×10^-38^ | 9.83 | (6.05-15.99) | 2.90×10^-20^ | 12.72 | (9.27-17.46) | 6.87×10^-56^ |
| Model_5_ | Ascending | Reference | | | Reference | | | Reference | | |
|  | Stable | 3.24 | (2.27-4.61) | 7.42×10^-11^ | 2.40 | (1.65-3.49) | 5.25×10^-6^ | 2.81 | (2.17-3.64) | 3.45×10^-15^ |
|  | Descending | 14.66 | (9.65-22.28) | 2.88×10^-36^ | 9.03 | (5.53-14.76) | 1.61×10^-18^ | 11.96 | (8.70-16.44) | 1.15×10^-52^ |
| Model_PS_ | Ascending | Reference | | | Reference | | | Reference | | |
|  | Stable | 3.08 | (2.02-4.69) | 1.53×10^-7^ | 2.89 | (1.83-4.55) | 4.89×10^-6^ | 2.99 | (2.20-4.07) | 3.80×10^-12^ |
|  | Descending | 7.19 | (4.61-11.21) | 3.22×10^-18^ | 7.90 | (4.77-13.10) | 9.03×10^-16^ | 7.49 | (5.37-10.46) | 2.70×10^-32^ |

Model_0_: univariate model without adjustment of covariates.

Model_1_: adjusted for age, gender, ethnicity, baseline platelet count, antiplatelet treatment, platelet transfusion, transfusion amount, malignancies, hematologic diseases, immune therapy, thrombotic diseases, thromboinflammatory diseases.

Model_2_: additionally adjusted for first ICU location, ARDS, sepsis, SOFA, APS-III, and supports within 24 hours (mechanical ventilation, vasopressor, and dialysis) upon model 1.

Model_3_: additionally adjusted for differential vital signs upon model 2.

Model_4_: additionally adjusted for differential laboratory results upon model 3.

Model_5_: additionally adjusted for differential comorbidities upon model 4.

Model_PS_: adjusted for all aforementioned covariates using the propensity score (PS) method.


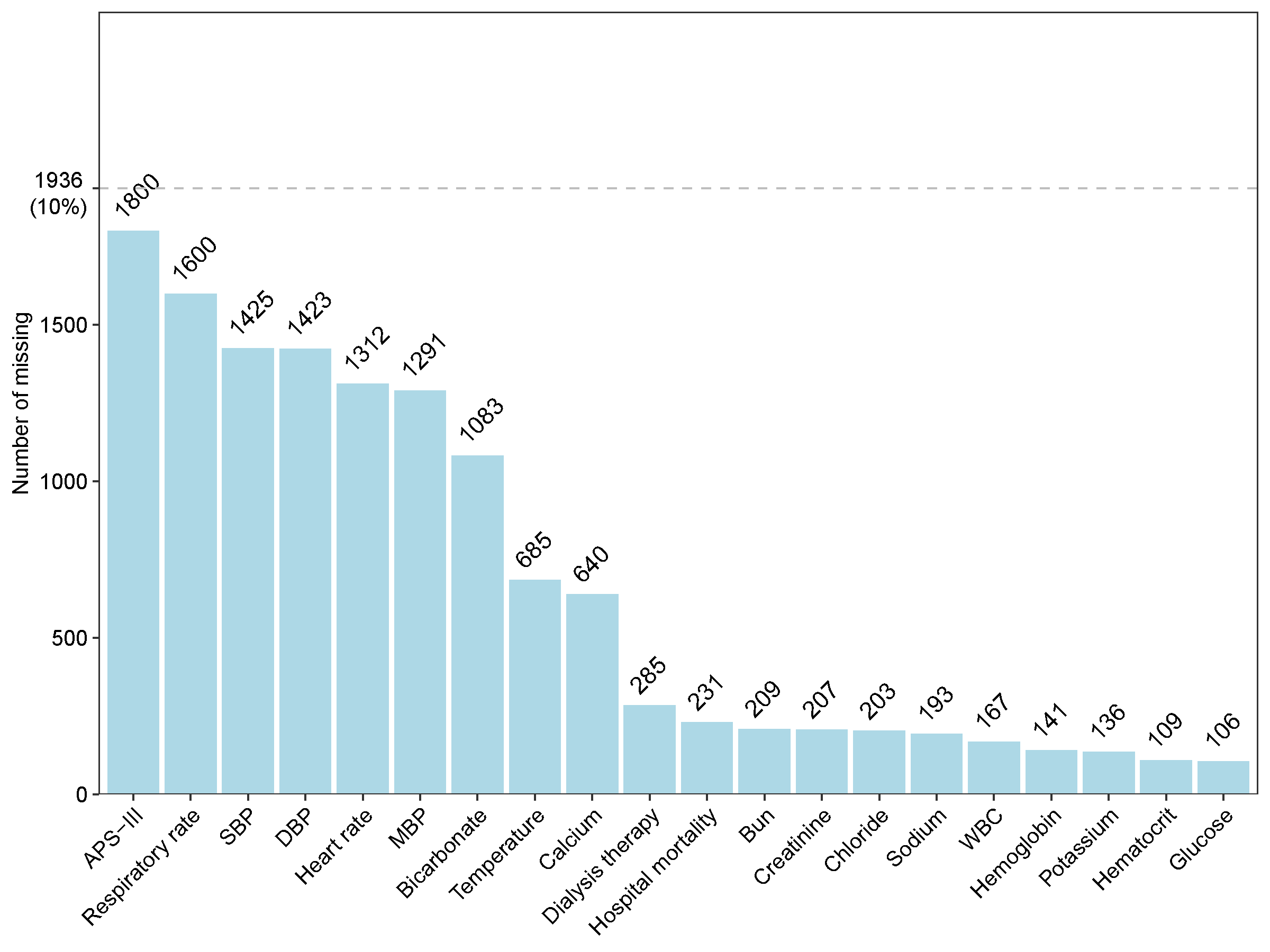


# **Supplementary Figure 1. Number of missing values of clinical variables in the eICU-CRD database**. APS-III: acute physiology score III; SBP: systolic blood pressure; DBP: diastolic blood pressure; MBP: mean blood pressure; BUN: blood urea nitrogen; WBC: white blood cell.


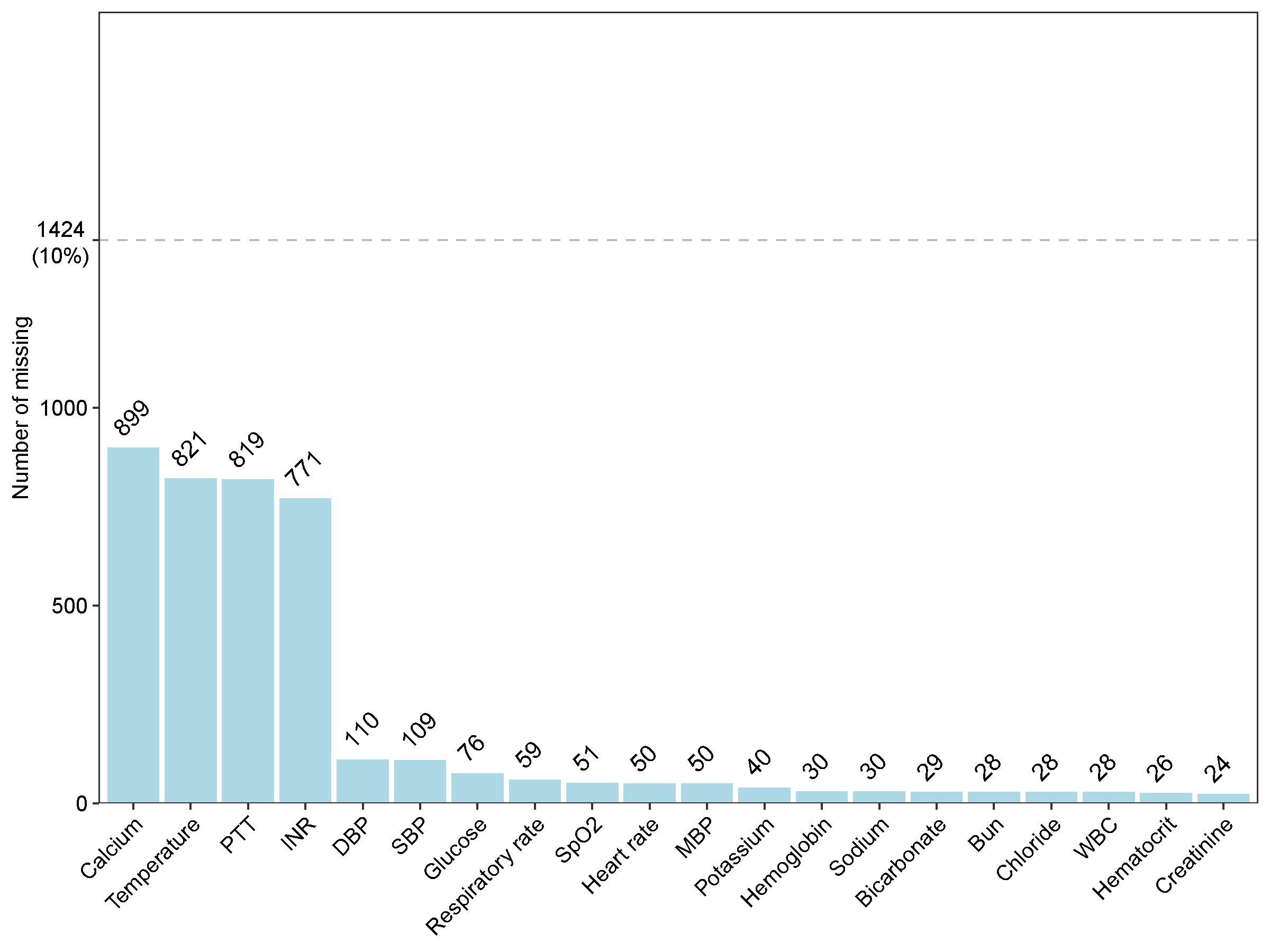


# **Supplementary Figure 2. Number of missing values of clinical variables in the MIMIC-IV database.** INR: international normalized ratio; DBP: diastolic blood pressure; SBP: systolic blood pressure; SpO2: pulse oxygen saturation; MBP: mean blood pressure; BUN: blood urea nitrogen; WBC: white blood cell.


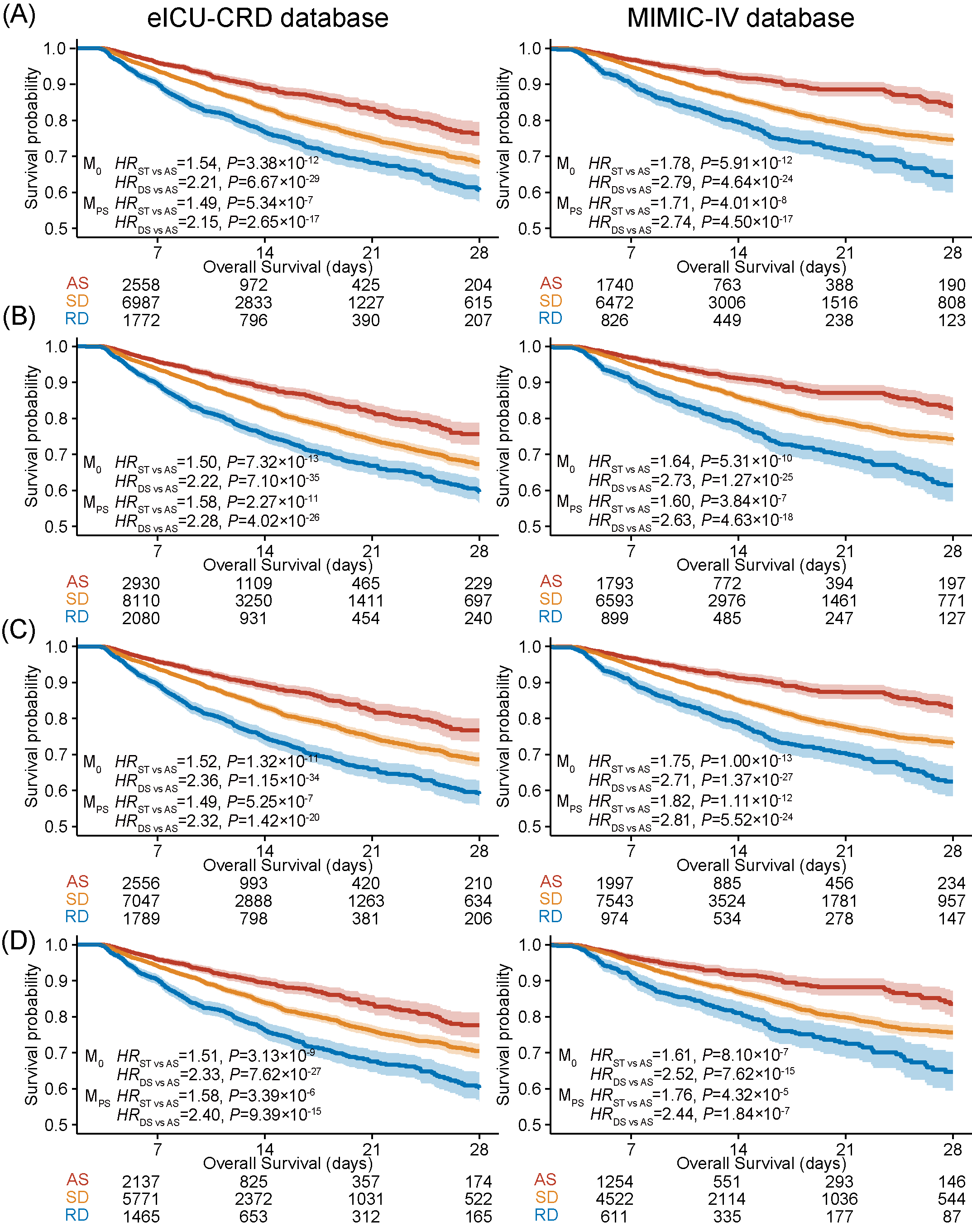


# **Supplementary Figure 3. Subgroup analyses for associations between longitudinal platelet trajectory and 28-day survival.** (A) Subgroup analysis by excluding patients with hematologic diseases, malignancies, or immune therapy. (B) Subgroup analysis by excluding patients with platelet transfusion. (C) Subgroup analysis by excluding patients with platelet transfusion or antiplatelet treatment. (D) Subgroup analysis by excluding patients with hematologic diseases, malignancies, immune therapy, platelet transfusion or antiplatelet treatment


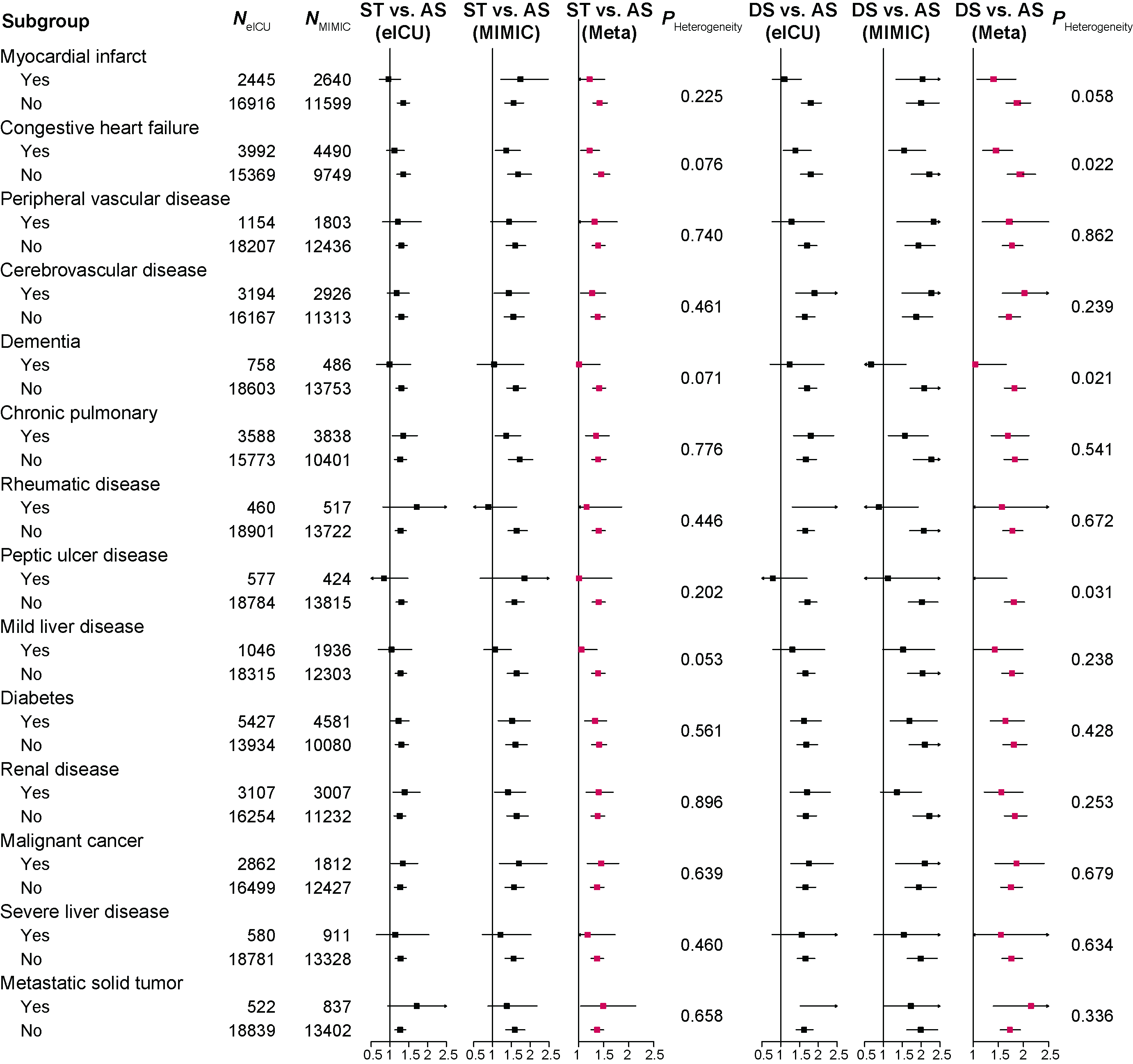


# **Supplementary Figure 4. Analysis of associations between dynamic platelet count trajectory pattern and 28-day overall survival stratified by comorbidities in the eICU-CRD and MIMIC-IV databases.** Meta-analysis was conducted to pool the results from eICU-CRD and MIMIC-IV databases.

**
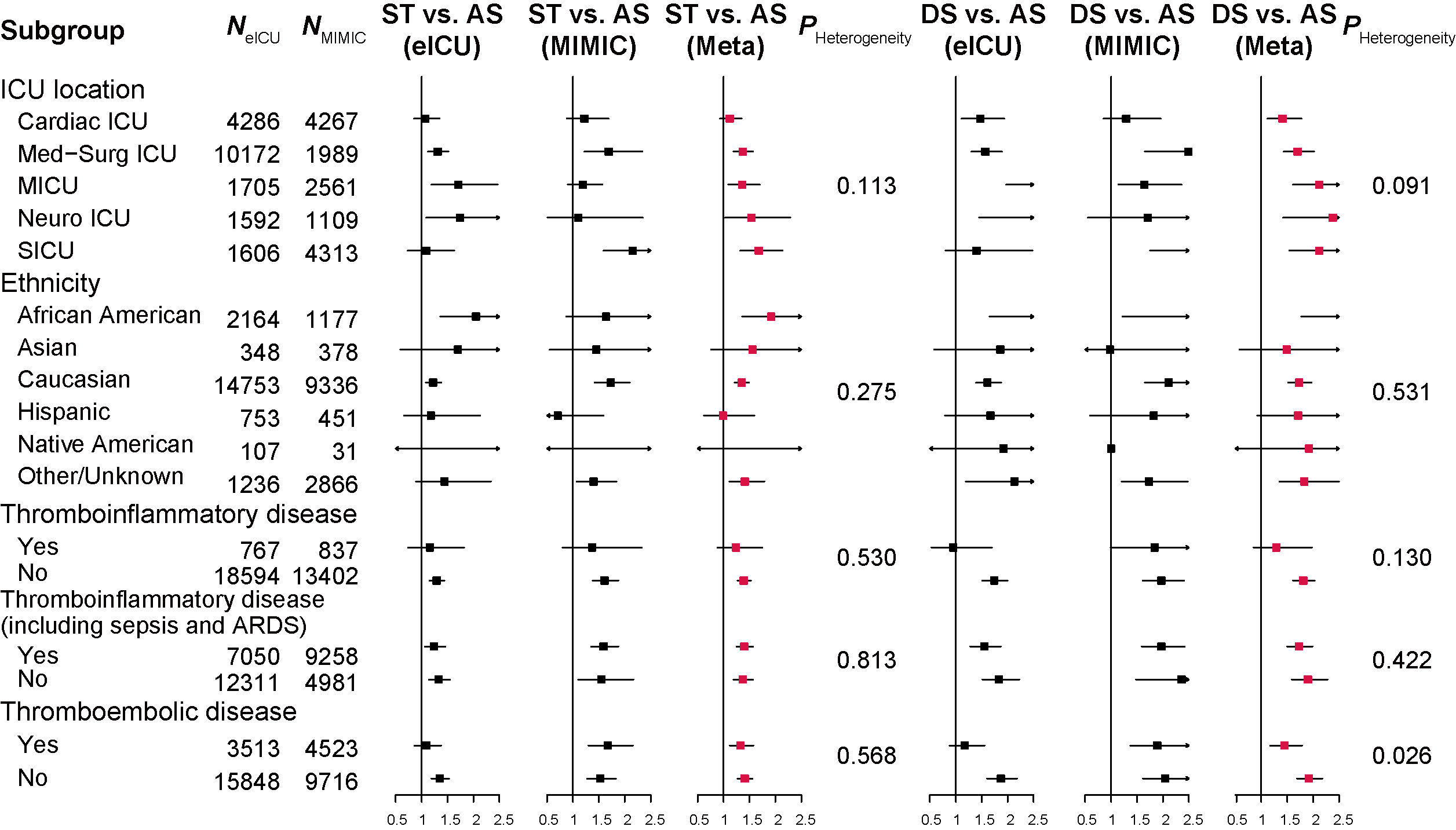
**

# **Supplementary Figure 5. Analysis of associations between dynamic platelet count trajectory pattern and 28-day overall survival stratified by ICU location, ethnicity, thromboinflammatory diseases, and thromboembolic diseases in the eICU-CRD and MIMIC-IV databases**


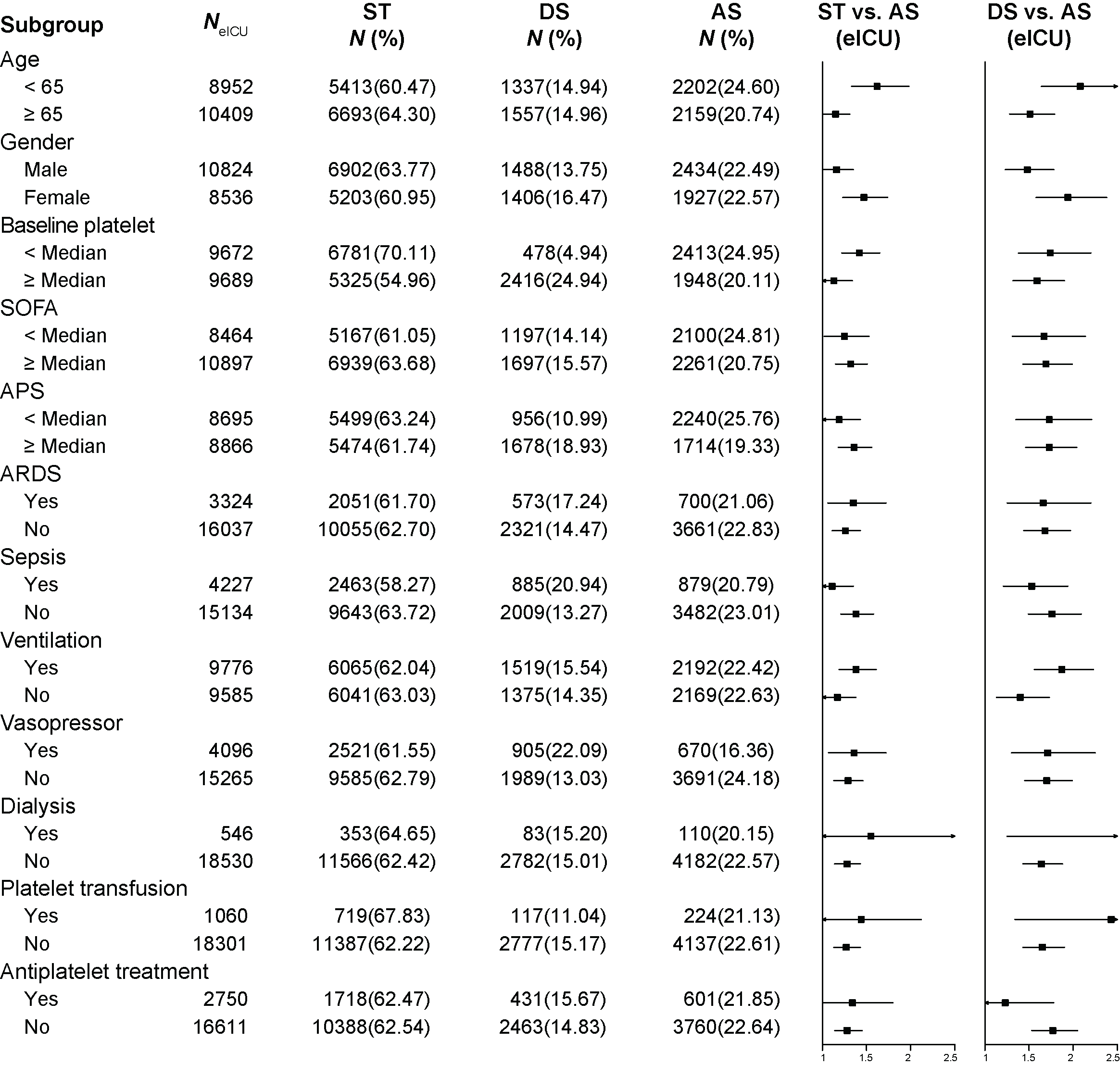


# **Supplementary Figure 6. Distribution of the three dynamic platelet count trajectory patterns stratified by baseline characteristics, severity scores, and treatment supports in the eICU-CRD database.** SOFA: sequential organ failure assessment; APS-III: acute physiology score III; ARDS: acute respiratory distress syndrome.


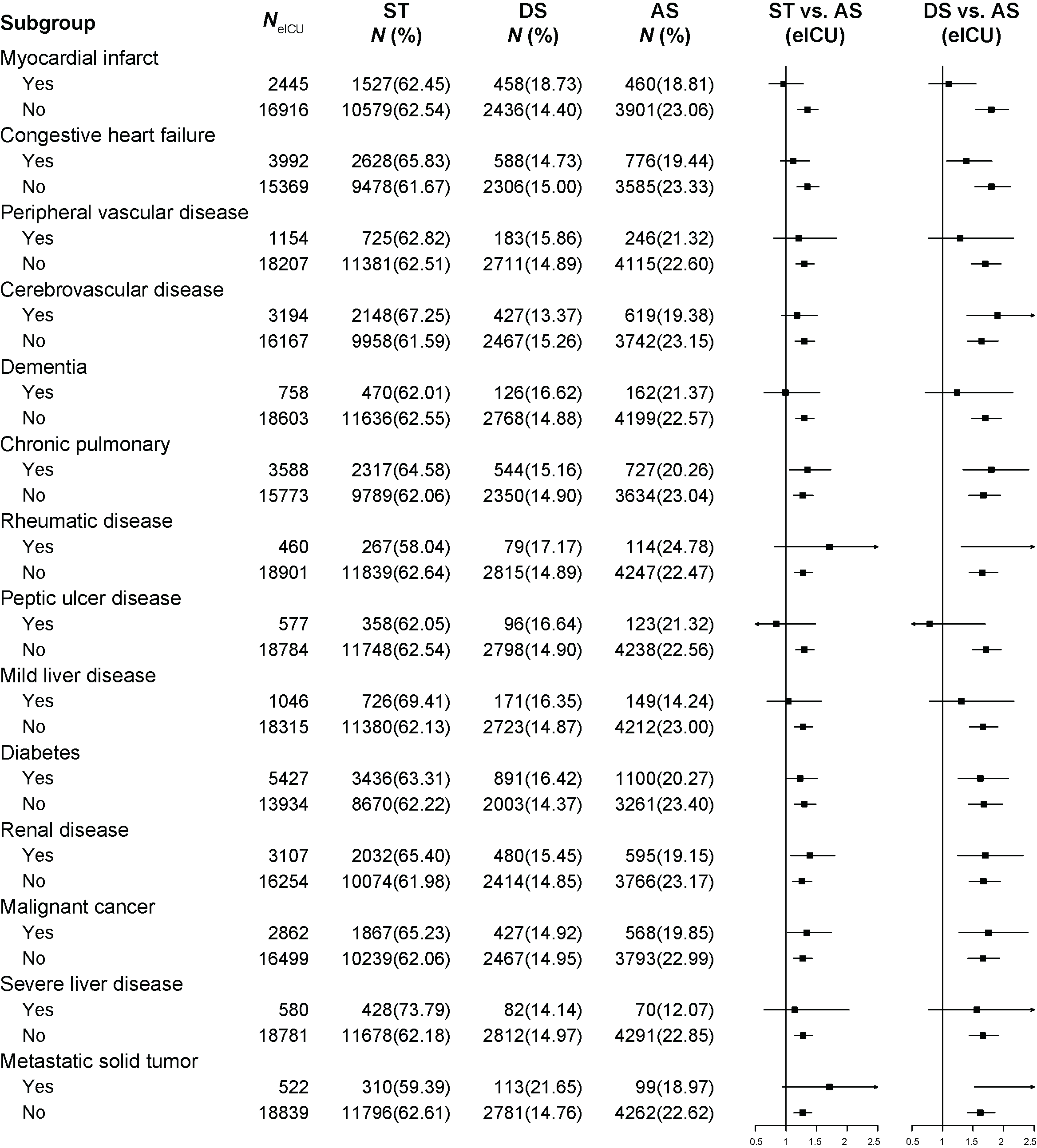


# **Supplementary Figure 7. Distribution of the three dynamic platelet count trajectory patterns stratified by Charlson comorbidities in the eICU-CRD database.**

**
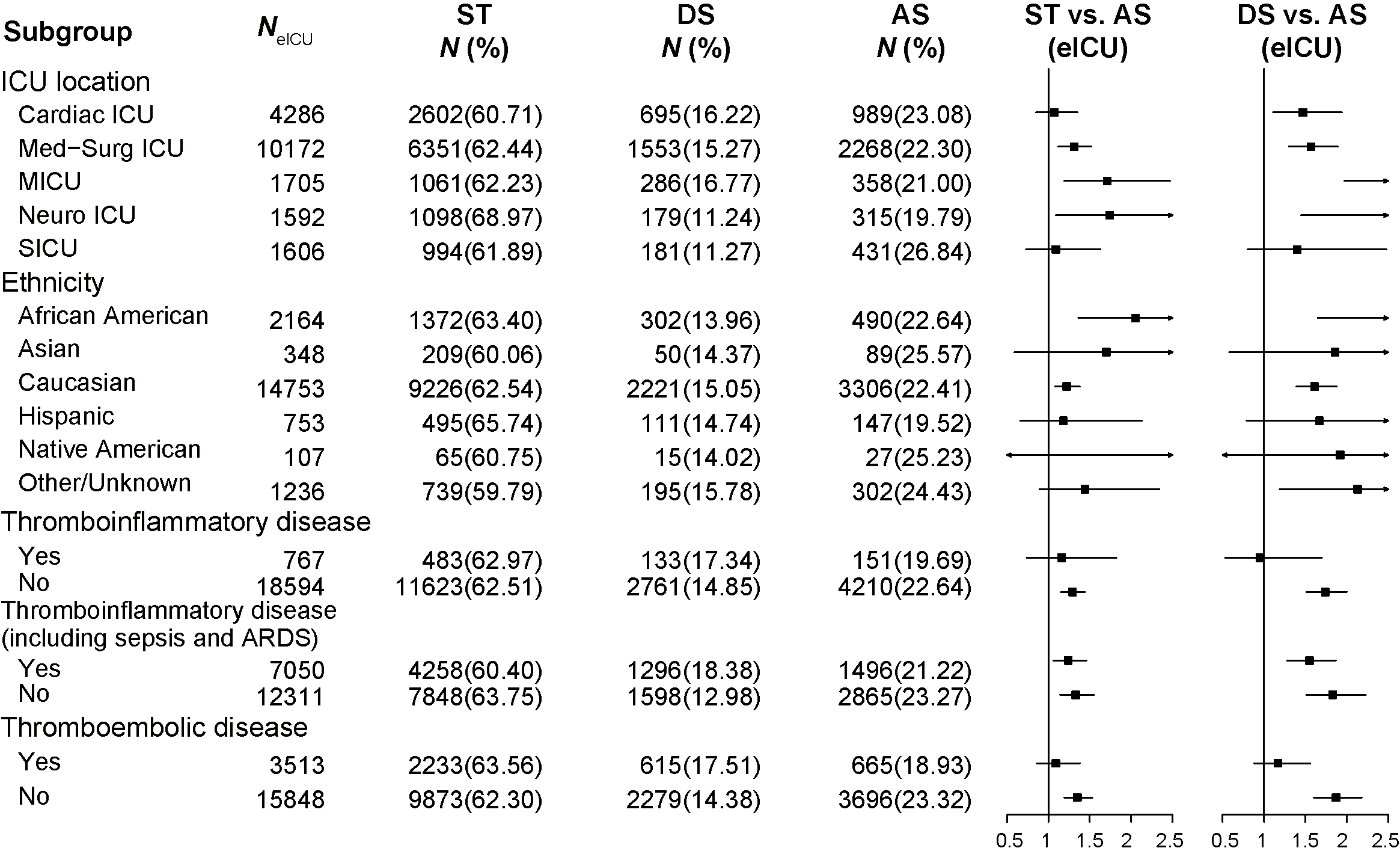
**

# **Supplementary Figure 8. Distribution of the three dynamic platelet count trajectory patterns stratified by ICU location, ethnicity, thromboinflammatory diseases, and thromboembolic diseases in the eICU-CRD database**


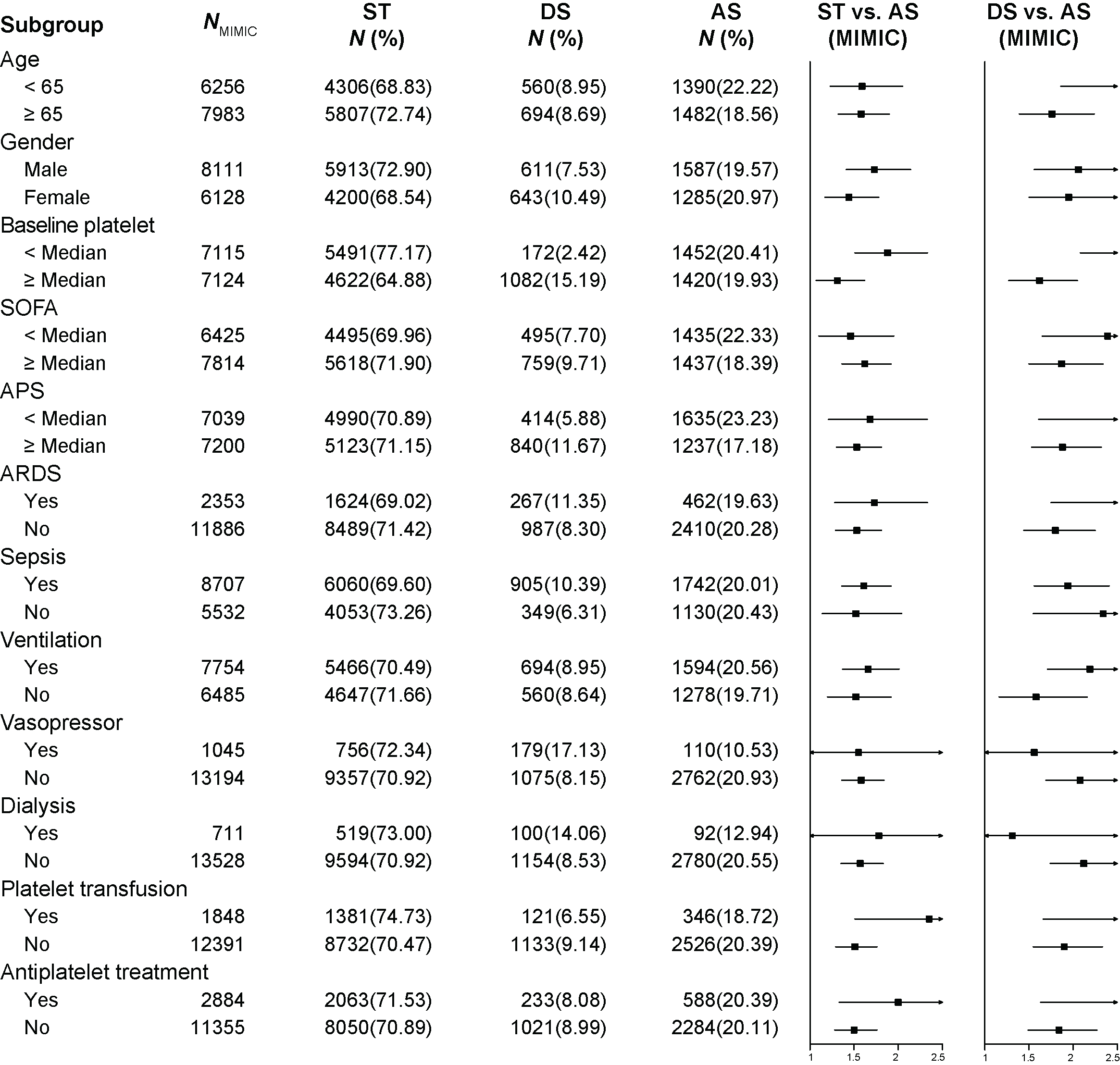


# **Supplementary Figure 9. Distribution of the three dynamic platelet count trajectory patterns stratified by baseline characteristics, severity scores, and treatment supports in the MIMIC-IV database.** SOFA: sequential organ failure assessment; APS-III: acute physiology score III; ARDS: acute respiratory distress syndrome.


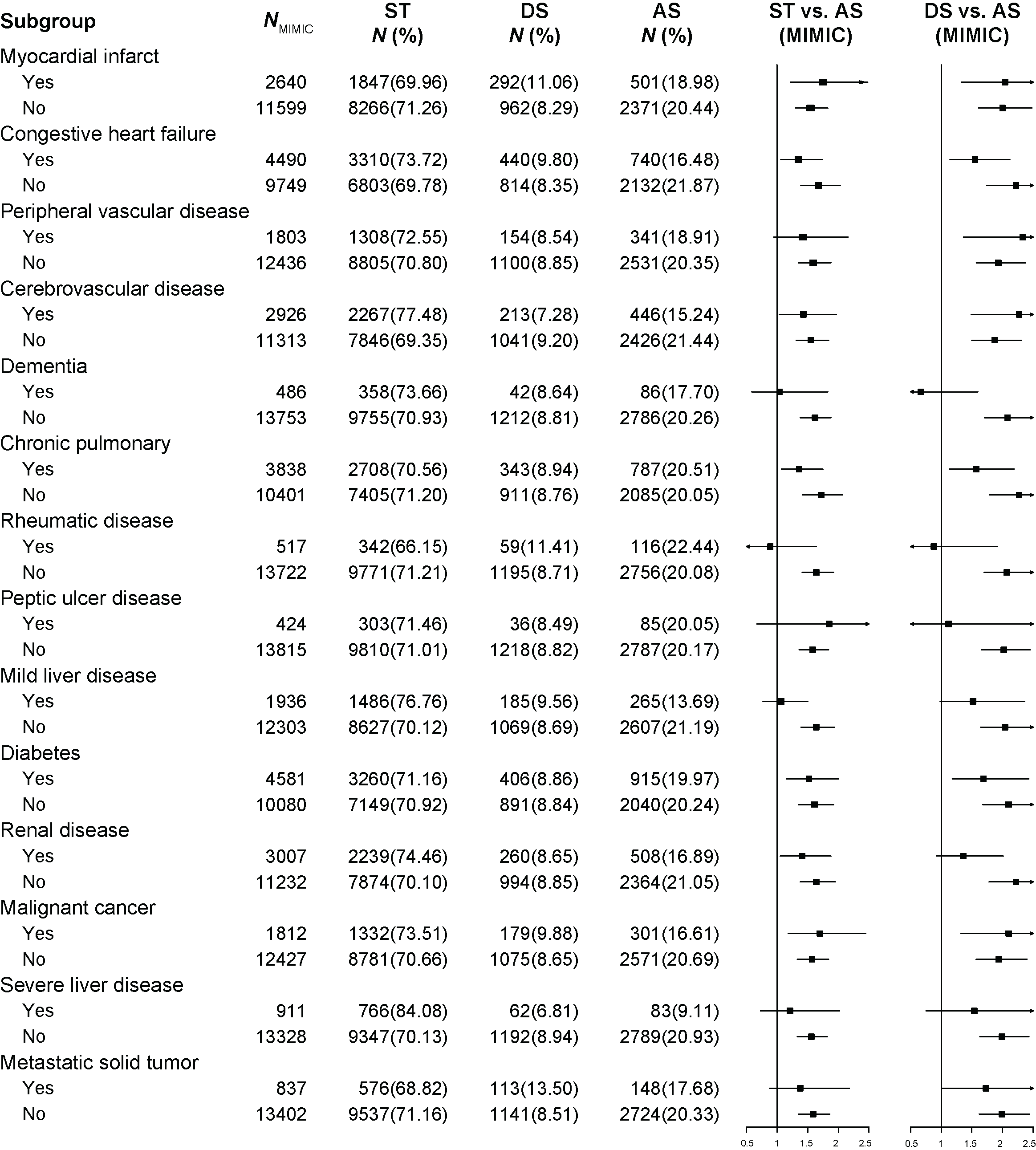


# **Supplementary Figure 10. Distribution of the three dynamic platelet count trajectory patterns stratified by Charlson comorbidities in the MIMIC-IV database**

**
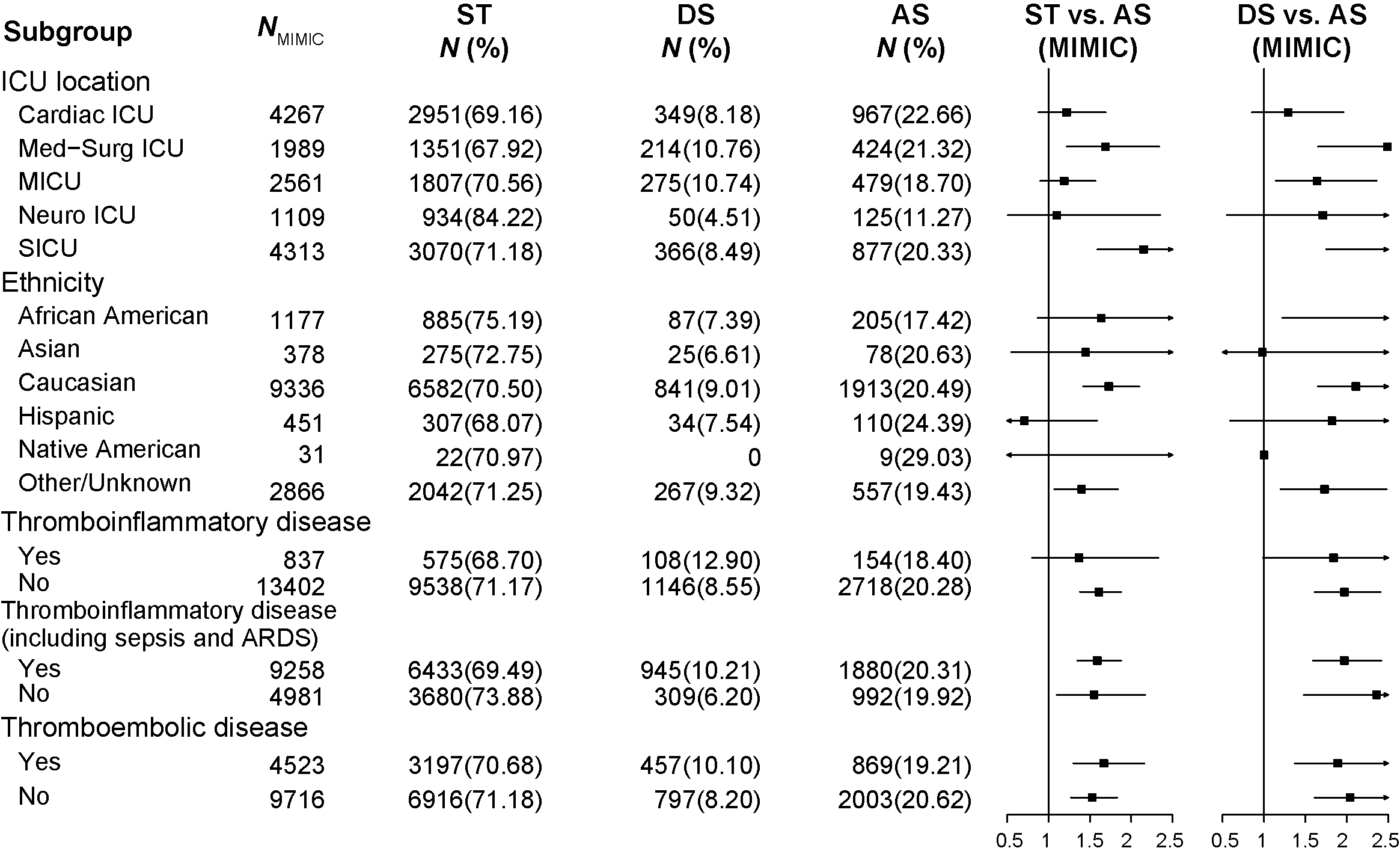
**

# **Supplementary Figure 11. Distribution of the three dynamic platelet count trajectory patterns stratified by ICU location, ethnicity, thromboinflammatory diseases, and thromboembolic diseases in the MIMIC-IV database**


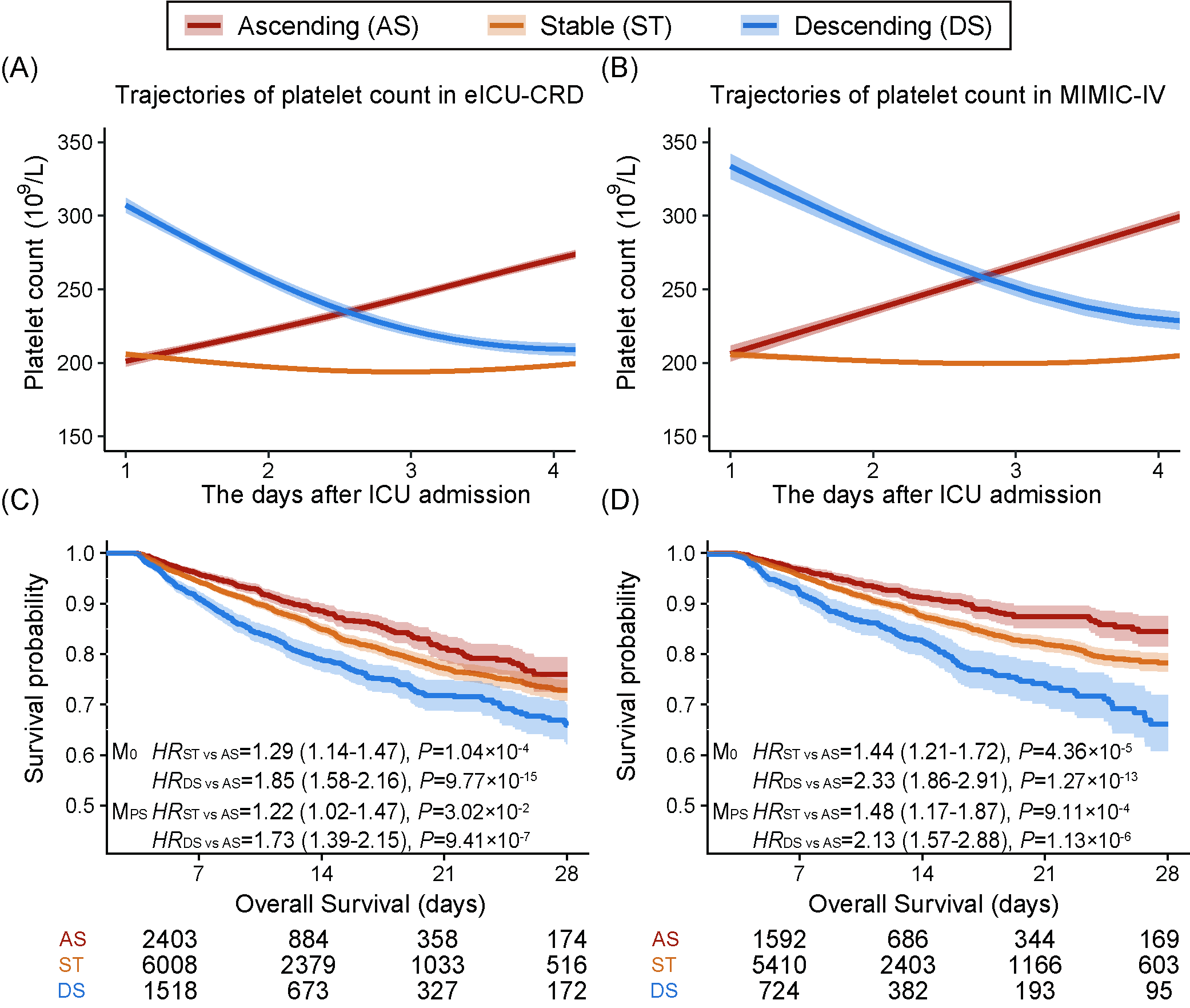


# **Supplementary Figure 12. Subgroup analysis of patients without thrombocytopenia during the entire ICU hospitalization.** M_0_: univariate model without adjustment of covariates. M_PS_: adjusted for age, gender, ethnicity, baseline platelet count, antiplatelet treatment, platelet transfusion, transfusion amount, malignancies, hematologic diseases, immune therapy, thrombotic diseases, thromboinflammatory diseases, first ICU location, ARDS, sepsis, SOFA, APS-III, and supports within 24 hours (mechanical ventilation, vasopressor, and dialysis), differential vital signs, differential laboratory tests and differential comorbidities using the propensity score (PS) method.

**
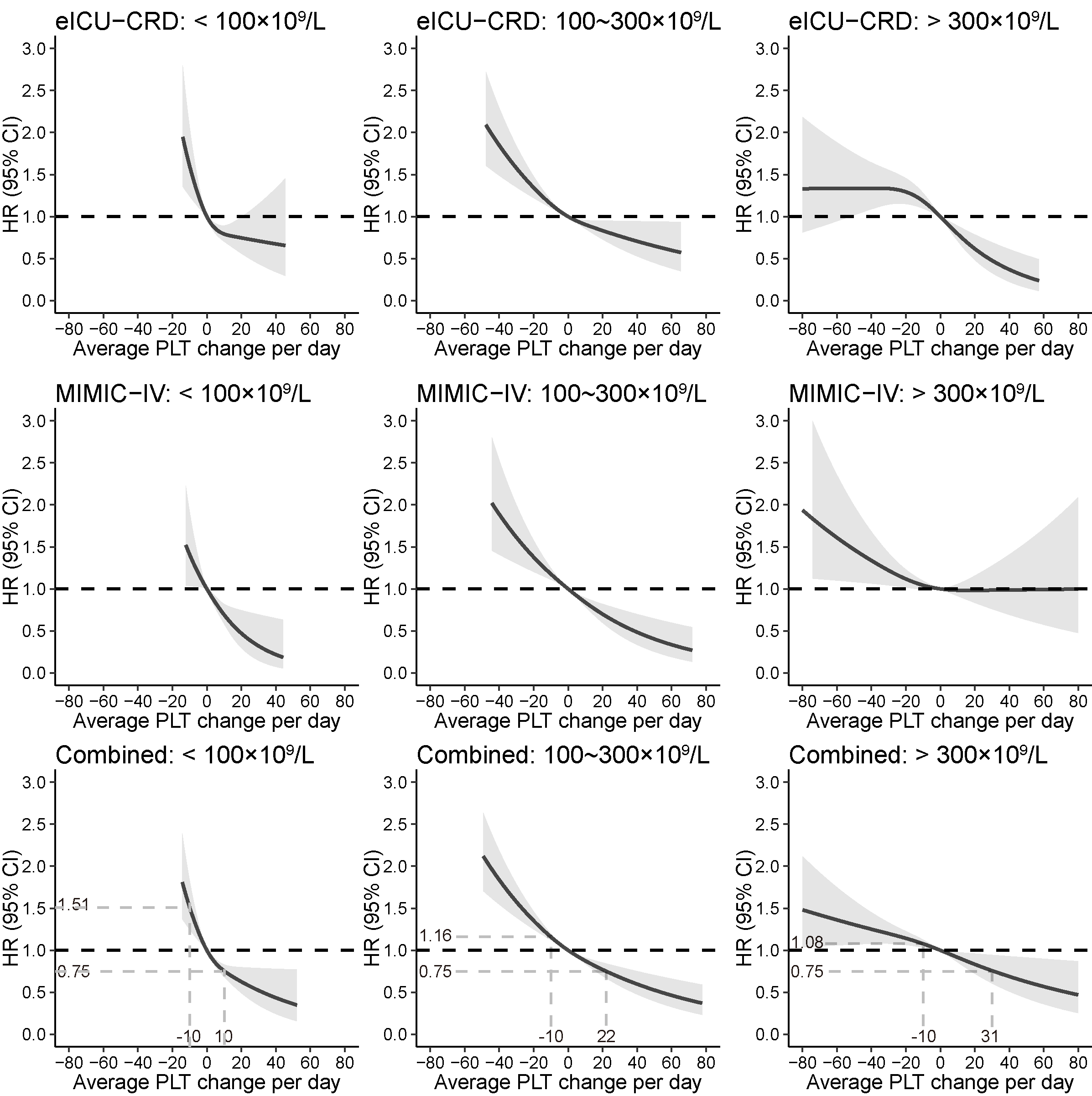
**

# **Supplementary Figure 13. Risk quantization of platelet count changes per day on 28-day mortality risk.** *HR*s and 95% CIs of platelet count changes per day were derived from restricted cubic spline regression and the shallow area represents 95% CI. Subgroup analysis stratified by normal range of platelet count (100-300×10^9^/L) is presented.


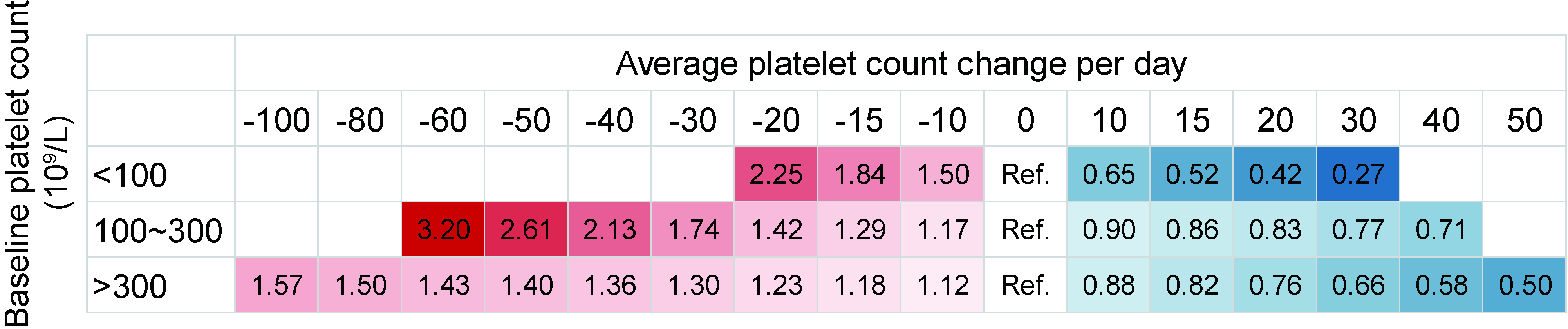


# **Supplementary Figure 14. Hazard risks estimated in the MEARDS cohort.** The values in each cell represent hazard risks of platelet count changes per day derived from restricted cubic spline regression using the MEARDS database.
